# Is the current regulatory framework for direct-to-consumer microbiome-based tests sufficient to protect consumers from medical, economic, and dignitary harms?

**DOI:** 10.1093/jlb/lsaf024

**Published:** 2025-12-24

**Authors:** Diane E Hoffmann, Felicia D Langel, Erik C von Rosenvinge, Francis B Palumbo, Mary-Claire Roghmann, Jacques Ravel

**Affiliations:** University of Maryland Carey School of Law, 500 W. Baltimore St., Baltimore, MD 21201, USA; Vaccine Injury Compensation Program, U.S. Department of Justice, Civil Division, Washington, D.C. 20002, USA; University of Maryland School of Medicine and the Veterans Administration Maryland Health Care System, Baltimore, MD 21201, USA; University of Maryland School of Pharmacy, 20 N. Pine St., Baltimore, MD 21201, USA; University of Maryland School of Medicine and the Veterans Administration Maryland Health Care System, Baltimore, MD 21201, USA; University of Maryland School of Medicine, 670 W. Baltimore St., Baltimore, MD 21201, USA

**Keywords:** diagnostic, direct-to-consumer, microbiome, regulation, tests, validity

## Abstract

This article offers a thorough analysis of an important public health issue–the lack of regulation of companies selling direct-to-consumer (DTC) microbiome-based tests. These companies invite curious consumers and desperate patients to submit stool and/or vaginal secretion samples to them for analysis. Purchasers receive a report about the composition of their gut and/or vaginal microbiomes with recommendations to change their diet or take certain dietary supplements. The piece is grounded in a study the authors conducted of DTC microbiome testing company websites and their practices, which are often misleading to consumers. Moreover, the tests lack analytical and clinical validity. This means they may have many “false positive” or “false negatives” and can harm consumers who rely on them as a basis for determining their health status The current regulatory framework for these tests has significant gaps. These include the lack of proficiency testing under the Clinical Laboratory Improvement Amendments (CLIA) and the lack of regulation by FDA as a medical device. Although FDA has distinguished DTC tests from other Laboratory Developed Tests and asserted that they may pose unique risks because they are ordered outside of a physician-patient relationship, they have largely ignored this group of tests, likely because they view them as low risk, general wellness tests and exempted from regulation as medical devices under the software exemptions in the 21st Century Cures Act (Cures Act). The authors conclude, however, that many of these tests are not low risk general wellness tests, nor do they meet the exemptions under the Cures Act and as a result should be more stringently regulated.

## I. INTRODUCTION

Researchers wanting to start direct-to-consumer (DTC) microbiome-based testing^1^ companies have attracted ‘hundreds of millions in venture capital funding’ and established ‘dozens of new startups.’^2^ This proliferation in commercial microbiome testing follows on the heels of DTC genetic testing^3^ and reflects growing consumer interest in personalized medicine. Gut microbiome–based testing particularly appeals to this interest because the gut microbiome has been associated with numerous medical conditions that may be improved through dietary and lifestyle changes.^4^ Similarly, consumers may pursue vaginal microbiome–based testing in order to detect ‘disruptive and protective bacteria/fungi that research has shown are related to’ cases of chronic vaginal infections.^5^ Relatively new to the marketplace are companies marketing DTC oral^6^ and skin microbiome tests.^7^ Recognizing a commercial opportunity, private companies are actively entering the market with offerings of these tests, which are moderately priced, provide laboratory results without the inconvenience of scheduling a medical appointment, and empower consumers with information about their microbiome and purportedly their health.^8^

The growth of the DTC microbiome-based testing market, however, raises some of the same concerns encountered with the emergence of DTC genetic tests, such as confidentiality of consumers’ health data, limited evidence that the tests can accurately inform clinical decision-making or dietary recommendations, dubious claims,^9^ and self-misdiagnosis when consumers rely on inaccurate results.^10^ Legislators and regulators wrestled for over a decade with how to address these concerns for genetic tests. Based on evidence of deceptive marketing and misleading test results^11^ and concerns about how third-party use of test results would affect genetic research, legislative and regulatory responses included new statutes, such as the Genetic Information Nondiscrimination Act (GINA)^12^ and similar state laws, as well as changes to the Food and Drug Administration’s (FDA) regulation of laboratory developed tests (LDTs).

With this new product class, ie DTC at-home sample collection kits and laboratory assessment of one’s microbiota, come new questions about whether the regulatory pathway for DTC genetic tests is a good fit for DTC microbiome-based tests or whether modifications to this pathway are necessary to adequately address the risks these tests may pose to consumers. The tests also share characteristics with at-home health tests that have skirted the line of medical device regulation because they fall into the category of ‘general wellness products,’ raising concerns about their relatively unregulated status.^13^ Additionally, the recent federal district court case, *American Clinical Laboratory Association v. FDA*,^14^ vacating FDA’s attempt to regulate LDTs, raises questions about the overall adequacy of the regulation of both DTC genetic and microbiome tests. A response to these concerns by federal legislators and regulators will need to consider whether consumers are adequately protected from harms to their health, safety, and privacy, as well as from financial exploitation, and, if additional regulation is necessary, how to implement it without stifling innovation in this nascent industry.

In this article, we review the rapidly growing DTC microbiome testing industry with a focus on gut and vaginal testing companies. We look at how these companies operate and the types of claims they make.^15^ Next, we introduce the relevant federal regulatory framework and describe how it has evolved to govern the related DTC genetic testing industry.^16^ Finally, we apply the current federal regulatory framework, including the impact of the *Am. Clin. Lab. Ass’n v. FDA* decision,^17^ to DTC microbiome-based tests and raise questions about whether they fit into the light-handed regulatory scheme that developed around DTC genetic tests, and, if not, whether additional or different regulation is required, including regulation in the areas of test validity and reliability, data privacy, human subjects protections, data discrimination, and forensic use of microbiome test results.^18^

## II. THE DTC MICROBIOME TESTING INDUSTRY

### II.A. The Market for DTC Microbiome-Based Tests and the Testing Company Business Model

The DTC microbiome-based testing market has been predicted to expand at a compound annual rate of 11.8 per cent from 2024 to 2032.^19^ The industry has its roots in the Human Microbiome Project (HMP), a National Institutes of Health research initiative that was funded from 2007 to 2016.^20^ The first phase of the HMP ‘characterized the microbial communities from 300 healthy individuals, across [five] different sites on the human body: nasal passages, oral cavity, skin, gastrointestinal tract, and urogenital tract.’^21^ The second phase addressed the function of microbial communities and their interaction with their human host.^22^ Both phases yielded improved laboratory tools that helped to shed light on the relationship of the microbiome to health and disease. Leveraging associations between composition and structure of the microbiome and disease outcomes, private companies soon commercialized the new tools in the form of DTC microbiome tests.

For this article, we conducted a thorough online search for DTC gut and vaginal microbiome testing companies. As of November 2024, our search yielded 30 companies operating in several countries/continents^23^: 23 in North America, 12 in Europe, three in Australia, two in India, and one in South Korea.^24^ All of the DTC microbiome testing companies we reviewed use a similar business model. Most companies analyze the gut microbiome, but an increasing number are targeting the vaginal microbiome, and a few provide testing for the skin or oral microbiome. For the most part, DTC microbiome-based tests are ordered online by consumers without the involvement of a physician.^25^

The tests typically consist of a kit containing a sterile swab and a plastic tube that the consumer uses to collect a small specimen (stool for gut test; vaginal secretions for vaginal test) to mail to the company for analysis. Most companies ask consumers to complete a questionnaire that includes demographics, dietary habits, health history, and, for vaginal tests, sexual practices. This information is referred to as ‘metadata.’ Tests cost between $100 and $300,^26^ and results are received in 2–4 weeks. The report often includes a graphical scorecard that, at a minimum, identifies the microbes present, compares these microbes to a consumer database, and, in the case of gut microbiome analysis, makes dietary and/or nutritional supplement recommendations with the intent of improving the consumer’s gut health. Consumers pay more for tests that measure an extended panel of analytes and for consultation with a company-affiliated health care professional, usually a dietitian or nutritionist. However, several companies offer consultation with either a personal health, microbiome, or ‘vaginal coach.’^27^ Some companies also offer membership or subscriptions.^28^

DTC microbiome testing companies use genetic techniques to analyze the microbiome, specifically, next-generation sequencing such as 16S rRNA gene amplicon sequencing or metagenomic sequencing. These sequencing techniques describe taxonomic composition of the microbiome by identifying which microorganisms are present in a specimen and their relative abundance.^29^ This may include pathogens as well as commensal bacteria. Companies may develop their own in-house tests based on these genetic techniques. Alternatively, they may rely on external laboratories to perform the analysis. The companies/laboratories use software (often combinations of open-source and custom) to analyze the resulting data to make health and lifestyle recommendations.

For the most part, DTC microbiome testing companies are not explicitly claiming that their tests can diagnose illness or inform treatment decisions, but some companies are using the data they collect from consumers to conduct research in-house, or they scour the literature and analyze the published data to learn how the gut microbiome relates to different health conditions.^30^ Some companies provide this literature to customers to buttress their conclusions. Additionally, a few companies are partnering with academic research laboratories and using the academic data to clinically validate their tests. Through this research, some of these companies may aim to obtain FDA approval or clearance to ultimately use their microbiome tests to diagnose disease and/or make treatment recommendations. Most DTC microbiome testing companies, however, enter the market with semi-proven data analytics that they attempt to validate by building a robust dataset over time that relies heavily on the clinical or research information collected from their early users.^31^ For example, the company might compare a consumer’s data with those in the database of patients with certain diseases^32^ or to those who are deemed ‘healthy’ or ‘normal.’^33^

Companies then make recommendations based on this comparison. For example, one company provides a report to consumers with scores in six metabolic categories, an interpretation of the scores, and a recommendation based on the interpretation for personalized dietary modifications and supplements.^34^ Another company provides test results and recommendations for a hypothetical consumer listing 63 vegetables the consumer should eat and five vegetables to avoid.^35^ The report explains that the recommended vegetables are unlikely to cause an increase in blood sugar and may improve digestive efficiency and intestinal barrier health. Avoiding the other vegetables may improve the consumer’s ‘sulfide gas production pathways.’^36^

These companies state that they analyze the composition of an individual’s microbiota to look for dysbiosis that may suggest an ‘unhealthy’ gut or vaginal microbiome. However, their claims of having the ability to detect unhealthy microbiomes are not fully substantiated by the literature and may lead to consumer exploitation by marketing expensive products and recommending repeat testing and probiotic use, which may not have any value.^37^ Until these tests can establish a verifiable link between one’s microbiome composition and a condition or disease, they cannot be used to diagnose disease and, more importantly, cannot inform clinical decision-making.^38^ Additionally, DTC microbiome testing companies that use their tests to make unsubstantiated claims regarding health and wellness may put consumers at risk of harm when their products do not deliver on their claims.^39^

### II.B. The Potential of Microbiome-Based Diagnostics and Limits of Currently Available Tests

While the commercial market is developing products that allegedly provide consumers with health and wellness insights, the academic community is focused on advancing microbiome-based tests for the actual diagnosis of disease.^40^ One area of intensive research is the development of microbiome-based diagnostic tools for cancer. A 2022 review article concluded that ‘there is vast evidence indicating specific bacterial taxa and their associated genetic loci in cancer development and progression’ and that ‘many studies have suggested an association between variance in human bacterial composition and cancer.’^41^ An example is variations in the microbiome of the female genital tract, which have been linked to ‘cervical cancer, but also sexually transmitted disease and bacterial vaginosis recurrence.’^42^ Additionally, ‘imbalances in the lung microbiota’ have been associated with lung cancer.^43^ Further academic research studies may pave the way for a future in which microbiome-based tests become a routine part of medical practice to (1) detect biomarkers for diagnosis, (2) analyze patient microbiota to predict treatment outcomes, (3) develop personalized treatment strategies, and (4) monitor treatment success using microbiome time-series analysis.^44^

Despite ongoing academic research, most microbiome-based diagnostic tests have limited analytical^45^ and clinical validity due to a lack of supporting data.^46^ Determining the analytical validity of these tests is challenging because there is no standardization across the field.^47^ Companies employ different pre-analytic and analytic laboratory techniques, leading to variability in test results. To establish clinical validity, each testing company must independently conduct well-powered clinical trials that demonstrate the ability of a company-specific process to diagnose a specific condition or disease.

## III. CONCERNS ABOUT DTC MICROBIOME TESTING COMPANIES

DTC microbiome tests appear to attract two types of consumers: (1) healthy consumers who are curious about their microbiome and (2) chronically ill consumers who order these tests to address their medical conditions.^48^ As part of an NIH-funded research grant, we conducted focus groups with representatives of four stakeholders: microbiome researchers, clinicians, patients who might purchase these tests, and consumers who had ordered them. The purposes of the focus groups were to

1) identify the … motivations and perceptions of patients currently engaged with microbiome-based diagnostic tests;
2) determine under what circumstances potential consumers would or would not order such tests;
3) identify the … practice variables and perceptions of providers ordering these tests for their patients or concerns they have about ordering such tests;
4) determine how well patients and providers understand the test results; and
5) elucidate potential legal or social implications of these [tests] that should inform their regulation.^49^

Participating patients included individuals from a national medical center who had been diagnosed with various gastrointestinal conditions and who had not ordered the tests but might be interested in such tests. Consumers included individuals who had taken the tests offered by the American Gut Project (now called the Microsetta Initiative), an open platform for citizen science microbiome research.^50^ When asked about the potential benefits of these tests, one healthy participant stated that she thought ‘the benefits would include a better understanding of which microorganisms you are lacking and which you may have an overabundance of.’ Another stated, ‘This particular type of test could be good for a baseline, [and subsequent tests could then reveal] how those numbers (high, low, and normal) have fluctuated over time.’ In describing why she got the test, a participant with a chronic illness stated, ‘I had been dealing with [an illness] for about 10 plus years, but I just reached my breaking point where I decided to go all in … and figure it out once and for all. Ultimately, it led to a fecal transplant…. So yeah, [this test] was like just a stop on the journey.’ Focus group responses such as these suggest that consumers may have unrealistic expectations of the tests and a misunderstanding (likely fueled by company claims) of what these tests can reliably tell them.

### III.A. Focus Groups with Gastroenterologists, Gynecologists, and Functional Medicine Physicians

Focus groups with specialty physicians who were most likely to be familiar with these tests confirmed the conclusion we drew from the consumer/patient focus group results that microbiome tests are likely to be misunderstood.^51^ These specialists included adult and pediatric gastroenterologists and gynecologists (hereinafter traditional providers), and functional medicine physicians.^52^ Functional medicine physicians were the most likely providers to have ordered these tests for their patients or suggested that their patients order the tests.^53^ In contrast, traditional providers did not order these tests, viewing them as difficult to interpret and not providing meaningful results.

Many traditional providers were concerned about these companies’ analytical procedures and their ability to detect variants in the microbiome, as well as whether the testing process was standardized across the DTC testing companies. Providers from both the traditional and functional medicine groups were concerned that these tests may provide recommendations to address a specific microorganism that may not have clinical relevance. Additionally, they asserted that addressing specific microorganisms by recommending supplements or elimination diets may be harmful and disrupt the complex, symbiotic nature of the microbiome, leading to worse health outcomes. They said this was of particular concern for DTC tests, where patients may implement these interventions without consulting a physician. In addition to potentially affecting health outcomes, some providers from both groups (ie traditional and functional medicine) said that focusing on specific microorganisms may delay the detection and treatment of the root cause of the health issue.^54^

A few traditional providers viewed these tests as ‘baseless products [intended] to take advantage of patients.’^55^ They further expressed concerns about the costs of the tests and associated ‘treatments,’ including probiotics, herbs, and dietary changes. Several also said that their patients come to them with their results after ordering the tests online for themselves or their children and ask for help with interpretation. A few pediatric gastroenterologists stated that it was parents of children with various gastrointestinal (GI) symptoms and no definitive cause that often order these tests, seeking answers to their children’s complex health issues. These physicians also shared that parents of children who have not shown improvement through conventional treatments, or who are on the autism spectrum, have ordered these tests. One pediatric gastroenterologist said:

It often is families that have had challenges in finding what they see as therapeutic relationships or diagnostic answers to their questions…. In addition to patients with irritable bowel, we see patients who have functional syndromes [including] functional abdominal pain, and also a number who have children who are on the autism spectrum…. Rates of GI diseases and GI symptoms, in particular, are higher in our autism population. Often these families really struggle with having children who have significant behavioral needs. And then on top of it [have] GI symptoms that range from belly pain to aggression to constipation to diarrhea. They'll often come in with these tests looking for answers.^56^

Gastroenterologists also expressed concern about some of the dietary recommendations the test results included. Pediatric gastroenterologists were particularly worried that, because of the test recommendations, their patients may restrict their diet too severely, leading to greater health issues. One said she has seen patients develop avoidant restrictive food intake disorder after following microbiome or food intolerance testing recommendations to avoid certain foods.^57^

Several gynecologists expressed concern about the accuracy of these tests, in particular results about microorganisms associated with sexually transmitted infections (STIs), given that very little information is provided about how these tests detect STIs. These providers also discussed the potential harms to patients of receiving false-negative results for STIs, as well as the difficulties interpreting the findings for their patients. Additionally, the gynecologists expressed concerns about false-negative test results for human papilloma virus (HPV) as patients who are actually HPV positive may think they are fine and not seek attention from their medical provider.^58^

During the focus groups, providers were given a set of “mock” test results modeled after several DTC microbiome test results available on the internet. They were asked to answer a set of questions based on their own experience reviewing these tests or on the mock test results. Some noted that, while typical laboratory results cite to ranges for what is considered ‘average,’ the mock results did not provide this information and the reports that did include ranges, did not state how ranges are determined and on which populations they are normed. This lack of clarity regarding ranges and associated algorithms contributed to their lack of trust in the results. One pediatric gastroenterologist stated, ‘I worry about how these tests were performed.’ An adult gastroenterologist said, ‘I would suggest there be some kind of standardization in the process of doing these tests, so that… if different labs are doing it, they’re using the same process and… results are comparable.”^59^

Several providers, particularly the gastroenterologists, suggested that the companies that manufacture these tests should provide data on how they determine analytical and clinical validity. The lack of this data also made it difficult for them to have confidence in the validity of the results. As one pediatric gastroenterologist stated, ‘I rarely find [the results] to be useful because… firstly, you don’t get much information about how they perform the test to see how valid they are.’ Another gastroenterologist explained the need to assess the predictive ability of these tests, stating, ‘they’d have to validate it, they’d have to do this in 1,000 patients and see if they can confirm that something was predictive.’^60^

Several participants across provider groups stated that our current understanding of the microbiome has not advanced to the point of understanding the complex criteria for a healthy microbiome, what is needed to prevent dysbiosis, or the relationship between specific bacteria and health conditions. Often, microbiome-based test results will focus on the presence of a few specific bacteria. With the current understanding of the microbiome, some providers said it is difficult to comprehend the impact of these bacteria in the context of the larger microbiome. Participants explained that, due to this limited understanding, it is not possible to use microbiome-based test results to guide treatment options.^61^

Providers across all specialty groups believed that there should be greater regulation of DTC microbiome tests. Several suggested regulating the use of these test results as patient health information, regulating test recommendations, and requiring standardization and transparency of test validation processes.^62^

### III.B. Historical Missteps: the Case of uBiome

The comments by providers in the focus groups cast a shadow on the legitimacy of DTC microbiome tests and some of the practices of these companies. For one early-to-market DTC microbiome testing company, uBiome, these concerns were warranted. uBiome, a market leader, was identified by law enforcement as engaging in insurance fraud and other illegal activities. The company, founded in 2012 by Jessica Richman, a computer scientist, and Zac Apte, a biophysicist, was the first to bring to market tests based on an alleged link between human health and the microbiome.^63^ uBiome marketed two clinical tests (ie SmartGut and SmartJane (an analysis of the vaginal microbiome)) that were available through a consumer’s health care provider^64^ and were covered by some health insurers. Additionally, the company marketed a microbiome test for the DTC market called Gut Explorer.

Richman and Apte obtained over $100 million in venture capital funds for their start-up, and, within 6 years, uBiome grew in value to $600 million. In 2018, uBiome made Fast Company’s list of the 10 most innovative companies in the world^65^ and, as a key player in the life sciences commercial market, ‘basically invented the category of the microbiome.’^66^ However, to facilitate the company’s rapid growth, uBiome engaged in several illegal activities including pressuring consumers through repetitive emails to submit multiple samples for testing and then billing the consumer’s private health insurer or Medicare ‘two or three times for the same set of tests.’^67^ Further, uBiome pressured its contract physicians to approve all test requests without establishing a doctor–patient relationship or determining that a given test was medically necessary.^68^

Usually, insurance companies flag vendors that repeatedly bill more than $500 without a clear medical reason,^69^ and health insurers began to deny uBiome’s billing claims. In April 2019, the FBI raided uBiome’s San Francisco offices and seized employee computers in an investigation of the company’s Medicare billing.^70^ Soon thereafter, Richman and Apte, as well as uBiome’s general counsel and its interim CEO, stepped down and uBiome filed for bankruptcy. When the company failed to secure a new buyer and lost its laboratory certification, uBiome ceased all operations and liquidated its business.^71^ By December 2019, uBiome had sold its testing patents and data, including personal health information that participants had given them, at auction to another company for less than 1 per cent of the company’s original value.^72^

On March 18, 2021, the Securities and Exchange Commission (SEC) charged Richman and Apte with ‘defrauding investors out of $60 million by falsely portraying uBiome as a successful start-up with a proven business model and strong prospects for future growth.’^73^ According to an SEC press release, the co-founders of uBiome ‘portrayed’ the company ‘as having a strong track record of receiving health insurance reimbursement for its clinical tests, which purportedly could detect microorganisms and assist in diagnosing disease.’^74^ The SEC, in its complaint, argued that these were false and misleading claims as the company’s income was dependent on ‘duping doctors into ordering unnecessary tests’ and, if the insurers had realized this, they would not have reimbursed the company for the tests. In addition to these charges, the U.S. Attorney’s Office for the Northern District of California filed criminal charges against Richman and Apte for their activities.^75^

While uBiome’s legal trouble stemmed from its fraudulent billing to Medicare and commercial insurers, the statement by the SEC that the tests were medically unnecessary speaks to the limited benefit of these tests. Although we believe very few, if any, DTC microbiome companies are now billing health insurers to cover the costs of their tests, we think there is reason to be concerned about the relative lack of regulation of these tests as compared to many other medical devices.

### III.C. Test Validity, Unsubstantiated Claims, and Harms Caused by Inaccurate Consumer Test Results

Health and wellness tests can harm consumers when the tests are not valid, companies make inaccurate or unsubstantiated claims about what the test results can tell consumers, and companies make recommendations for changes in behavior or diet that are not proven. As to the first of these, DTC microbiome tests currently on the market lack both analytical and clinical validity.^76^ Microbiome tests differ from genetic tests where the goal is to find a single biomarker, eg a gene mutation.^77^ In the microbiome testing context, the objective is to identify (1) all the microorganisms in a sample and (2) the amount or relative abundance of each. The prior is like finding a needle in a haystack, whereas the latter is analogous to identifying each strand of straw in the stack, categorizing each by color or size, and indicating the percent of each category in the stack. At this point in time, such reporting is not possible as scientists have not yet identified all the microorganisms that exist in the gut or vaginal microbiome as ‘the custom bioinformatic tools used do not identify or quantify all bacteria in a sample.’^78^ Even if all microorganisms were known, the tests would still likely lack analytical validity, or at least a way to discern it, as there is no reference standard by which to compare the findings of each laboratory’s assay. Without such a standard, we cannot know if organisms are missing or over-represented.

Currently, most companies compare their findings of the composition of a consumer’s sample to others in their consumer database, which is not likely to be representative of the general population. Such databases are limited.^79^ To supplement their in-house data, companies may ‘purchase patient data from researchers who have characterized the microbiomes of individuals with certain chronic conditions. In either case, the company is relying on nonrepresentative data generated with unknown computational techniques.’^80^

To establish accuracy, companies must be able to replicate their results from the same sample. A recent study by researchers at the National Institute of Standards and Technology (NIST) found that, for a number of DTC microbiome testing companies, this was not the case.^81^ The researchers sent three separate samples from the same fecal composite sample to each of seven different testing companies. The results indicated not only a lack of consistency across companies, but also within the same company.^82^

As to clinical validity, while human diseases are increasingly ‘linked with an altered or “dysbiotic” gut microbiota,… whether such changes are causal, consequential, or bystanders to disease is, for the most part, unresolved.’^83^ In addition, to tell a consumer that their microbiome is healthy or unhealthy requires comparison to a standardized reference of a healthy microbiome. Currently, there is lack of agreement among scientific experts as to what constitutes a healthy or unhealthy microbiome.^84^

Despite the shortcomings of the current state of the science, many of the DTC microbiome testing companies that we reviewed make claims on their website that state or imply that the tests can tell a consumer whether their microbiome is causing their current symptoms or is related to a disease, health condition, ‘healthy gut,’ or healthy microbiome. While the companies also state that their tests are not diagnostic, their claims may lead consumers to think otherwise.

Consumers are likely to incorrectly believe that these tests are regulated by the FDA when, in fact, ‘they [have until recently] straddled the line between regulated medical devices (if making diagnostic claims) and virtually unregulated health and wellness products’.^85^ As we noted in a prior publication,

the lack of regulation of these tests may put consumers at risk of harm when they rely on inaccurate test results and clinically unproven nutritional or food supplement recommendations. These harms may include self-misdiagnosis, delay in seeking medical treatment, and substituting nonmedical supplements for prescription medications. Many individuals who seek out these tests have serious chronic illnesses and are desperate to try anything that may mitigate their pain and suffering.^86^

Due to the lack of regulation of these tests, they are not subject to adverse event reporting. Thus, there is no centralized database of reports of cases where consumers may have been harmed by following the recommendations included with their test results.

## IV. ADEQUACY OF REGULATION OF DTC MICROBIOME TESTING

In determining the adequacy of the current regulatory framework for DTC microbiome-based tests, we consider several parameters: (1) effectiveness/accuracy of the test, (2) safety, (3) accuracy and sufficiency of consumer information, and (4) data privacy and potential harmful use of information by third parties. The efficacy or accuracy of these tests, along with their safety, is arguably the feature of most importance for regulation. If not accurate, consumers are paying for tests that have no value, and they may take action that is harmful and based on unreliable results. For non-invasive diagnostic tests, safety is linked to accuracy as it relates to the tests’ false-positive and false-negative rates and how that information is used by the consumer or patient. Consumer information is the information provided by the company, including claims made on its website and other marketing materials about the benefits of the test and how the information will be used by the company. Finally, data privacy relates to whether the company will provide the consumer’s personal information to third parties and the potential harms that could come to the consumer from outside parties having access to their data. In this section, we address how each of these factors are currently regulated for DTC health tests and how well those regulations apply to DTC microbiome tests.

### IV.A. Regulating Accuracy and Safety of DTC Health Tests

The Centers for Medicare and Medicaid Services (CMS) and FDA both potentially have a role in regulating the accuracy and safety of DTC microbiome tests. Since DTC microbiome testing companies handle human specimens and run laboratories that analyze the specimens, these microbiome testing companies, like other DTC health tests, are subject to the Clinical Laboratory Improvement Amendments of 1988 (CLIA) overseen by CMS.^87^ To the extent that DTC health tests are considered diagnostic, they would be subject to FDA regulations governing ‘medical devices’^88^ and/or to FDA guidance on ‘software as a medical device’^89^ unless exempt. Under the software guidance they may be exempt from medical device regulation as low-risk tests for general wellness and/or as tests for clinical decision-making support. Additionally, the 2025 *Am. Clin. Lab. Ass’n v. FDA* case, discussed *infra*, has interjected some uncertainty into FDA’s future regulation of DTC health and microbiome tests under medical device regulations.^90^

#### IV.A.1. CLIA and its Regulatory Gaps

CMS enforces CLIA to ensure quality testing in US laboratories.^91^ CLIA certification is mandatory for all laboratories that test ‘materials derived from the human body for the purpose of providing information for the diagnosis, prevention, or treatment of any disease or impairment of, or the *assessment of the health* [emphasis added] of, human beings.’^92^ Under CLIA, LDTs^93^ that have not been cleared or approved by FDA must be analytically validated in the laboratory’s own environment before results using the test can be released.^94^ Furthermore, CLIA regulations do not distinguish between facilities performing DTC testing and facilities providing testing that is ordered by a clinician.^95^ CLIA requires that a covered laboratory verify the analytical validity,^96^ but not the clinical validity, of its tests.^97^

CMS issues different types of certification to laboratories based on the level of complexity of their tests, ie high, moderate, or low.^98^ Any test that is moderately or highly complex must meet CLIA standards for quality control and assessment, and personnel, and the laboratory performing the test likely needs to enroll in a proficiency testing program.^99^ The methodological requirements are to ensure consistency across laboratories, while proficiency testing ensures that an individual laboratory’s results are consistent with an external reference standard.

The CLIA certification process can be time consuming and highly particularized. To achieve certification, laboratories must document and implement auditable tracking for all aspects of their operation and submit to an inspection that occurs at random and is repeated every 2 years.^100^ Furthermore, high-complexity tests have more stringent personnel requirements under CLIA, while simple tests with a low risk of producing an incorrect result may obtain a waiver from CLIA requirements.^101^ While CLIA requirements appear robust, there are shortcomings in the oversight process. For example, the analytical validation may not be verified by CMS until after the laboratory has already started marketing its test.^102^

Although some of the DTC microbiome testing companies we identified for this article provide disease information, many provide only health and general wellness advice. However, this still brings them within the umbrella of CLIA, as CLIA certification is required for all laboratories that provide information used to assess the ‘health of, human beings.’^103^ Furthermore, DTC microbiome-based tests would likely be considered high complexity by FDA because they employ genetic analysis (considered high-complexity tests),^104^ and a CLIA certificate of waiver does not apply to such tests.^105^

Eleven of the 23 DTC microbiome testing companies we reviewed that sell their product in the USA noted on their websites that they are CLIA certified or contract with CLIA-certified laboratories. It is unclear from our research whether the remaining US companies attempted or achieved CLIA certification. If a DTC microbiome testing company has the resources, CLIA certification can confer a competitive advantage by indicating to consumers that the company’s tests meet the federal standards for commonly used laboratory procedures and techniques.^106^ However, we have reason to doubt that CLIA certification of DTC microbiome-based tests demonstrates their analytical validity.

The CLIA standards for analytical validity do not explicitly address microbiome tests,^107^ and there are currently no CLIA-approved proficiency testing programs for these tests, likely due to the difficulty of confirming their analytical validity. Unlike other diagnostic tests that target certain biomarkers, as discussed *supra*,^108^ it is unclear whether microbiome-based tests can accurately assess what they purport to measure. Fundamentally, microbiome tests represent a paradigm shift in the way laboratory ‘testing’ has been performed. This is the case for a few reasons. First, the tests focus on the relative abundance of microorganisms in a sample, rather than detecting a specific gene, bacteria, fungi, or mutation. Second, in traditional diagnostic testing, the target is clearly known in advance, eg *Staphylococcus aureus*, and standards can be developed to validate the test. In the case of microbiome-based tests, the test must ‘discover’ the composition of the sample, making the tests much more difficult to validate as we do not know *ex ante* what the sample contains.

#### IV.A.2. Limits of FDA Medical Device Regulation

While CMS jurisdiction under CLIA does not extend beyond assessing analytical validity, FDA evaluates both the analytical and clinical validity of products but only if they are subject to the Agency’s premarket submission process. Of potential relevance to DTC microbiome-based tests, FDA oversees the regulation of (1) medical devices, (2) software as a medical device (SaMD), and (3) medical device marketing claims and misbranding.

Diagnostic tests potentially fall into the regulated category of medical devices. The Food, Drug, and Cosmetic Act (FD&C Act), section 201(h) defines a medical device in part as ‘an instrument, apparatus, implement, [or] machine,… which is… intended for use in [among other things] the diagnosis of disease or other conditions.’^109^ FDA regulates medical devices using a three-tiered classification system based on the intended use of the product and its level of risk to consumers. The higher the risk, the higher the classification and the more regulatory control FDA exerts over the product. For Class I devices, the lowest risk classification,^110^ only general regulatory controls apply. These are provisions that control for adulteration, misbranding, labeling, and good manufacturing processes.^111^ For Class II devices, general and special regulatory controls apply.^112^ For Class III, the highest risk classification, general controls apply and a premarket approval (PMA) application for the product containing data from non-clinical laboratory studies and clinical investigations must be submitted to FDA.^113^ Of the three medical device classes, only Class II and III products require a submission to FDA prior to marketing.^114^

Diagnostic tests in which specimens are taken from the body for external testing are referred to as *in vitro* diagnostic tests (IVDs).^115^ Like other diagnostic tests, IVD test safety relates to the likelihood of a false-positive or false-negative test result and the impact on the consumer/patient of such inaccurate results.^116^ A test that produces many false negatives may cause a consumer’s serious or treatable disease to be missed, whereas a test that produces many false positives may cause a consumer needless worry and costly follow-up testing or treatment for a disease that they do not have. IVD tests can include those based on next-generation sequencing^117^ and are classified using the same three-tiered risk-based system as other medical devices. Many IVDs fall into the Class II category because they may produce incorrect test results, incorrect interpretation of test results, or incorrect understanding of the test system.^118^

Class II devices require a premarket notification to FDA under section 510(k) of the FD&C Act.^119^ In a 510(k) submission, a company must demonstrate that its product is substantially equivalent to a previously FDA-approved product already on the market, called the ‘predicate.’ Historically, the predicate device was to relate back to a device marketed prior to 1976 when the medical device amendments were passed. Today, as the quality and designs of medical products have changed over time, the predicate device is the most recent 510(k) medical device approved by FDA for that particular product.

Alternatively, a company may request that a device with no substantial equivalent be classified as a Class I or II device using a *de novo* application.^120^ The *de novo* application includes an explanation of why the device would be safe and effective if general and special controls are applied, as well as clinical and non-clinical data demonstrating safety and effectiveness.^121^ If a company can successfully demonstrate either equivalency or safety and effectiveness with general/special controls, the device is ‘cleared’ or approved for marketing by FDA.^122^ Class III devices, in contrast, require a more comprehensive PMA submission to FDA wherein a company must prove that its product is both safe and effective based on clinical trials.^123^

#### IV.A.3. Exemption of Laboratory Developed Tests from Medical Device Regulation

LDTs, a type of IVD, have traditionally *not* been reviewed by FDA prior to marketing as a matter of policy.^124^ Although FDA consistently claimed to have the authority to regulate LDTs as medical devices if they were prescribed by a health care provider, it generally did not do so, instead applying its policy of enforcement discretion. This policy, however, was fraught with controversy in light of changes in the type of LDTs that became available over time and the possibility of harms based on false-negative or false-positive test results.^125^ As a result, in April 2024 FDA announced in a rulemaking that it would regulate LDTs as medical devices.^126^ This decision was subject to widespread criticism within the laboratory testing community and, not long afterwards, the decision was challenged by the American Clinical Laboratory Association (ACLA) and the Association for Molecular Pathology. On March 31, 2025, the U.S. District Court for the Eastern District of Texas vacated the Agency rule in its entirety, concluding that it exceeded the Agency’s statutory authority. The Court emphasized that ‘“devices” under the FDCA “are articles of commerce, not the kinds of services performed by doctors and laboratories,”’ and that Congress specifically addressed the regulation of clinical laboratories and their services through CLIA, an independent statutory framework.^127^

The FDA did not appeal the decision and on September 19, 2025, the FDA released a final rule rescinding its 2024 regulation that had classified LDTs as medical devices subject to FDA oversight. This leaves the regulation of LDTs by FDA unlikely in the near term unless the Agency interprets the decision in a way that allows it to continue to regulate some LDTs, such as DTC tests, or Congress steps in and provides explicit authorization for FDA to regulate them. As to the former, although many believe FDA pushback to the Court’s ruling is unlikely during the current Trump administration,^128^ there is at least one argument FDA could make in issuing revised regulations or guidance or taking enforcement action. The Agency could argue that, at a minimum, it continues to have the authority to regulate DTC tests as the definition of LDTs in the Court’s opinion is quite narrow. The Court defines the tests as ‘in-house diagnostic tests developed, validated, and performed by trained professionals within a single clinical laboratory. They are performed on specimens at the request of an individual physician, in the context of a specific doctor-patient relationship.’^129^ This definition clearly does not include DTC tests ordered by consumers over the internet without the involvement of a physician.

Some critics have argued that FDA could also attempt to use the 21st Century Cures Act to argue that Congress assumed when it passed the Act that FDA had the authority to regulate tests that rely on software as medical devices, even if they were LDTs. That assumption is why Congress needed to pass the Cures Act, carving out certain exceptions to the existing law at the time. These exceptions included tests using software for general wellness and for some other types of software-based tests. Using this rationale, FDA could regulate software-based LDTs as medical devices under revised regulations; however, such action would be highly unlikely at this time.^130^

As to congressional action, a number of commentators think this is unlikely any time soon. Congress considered such legislation in the recent past. In 2018, members of Congress introduced the VALID Act, which would have explicitly given FDA the authority to regulate LDTs using a risk-based framework similar to the one it applies for medical devices.^131^ The legislation did not pass, however, and was reintroduced in subsequent years, without success, despite the support of several medical device advocacy organizations.

Of potential relevance, in its Final Rule for the regulation of LDTs, consistent with the District Court’s definition of LDTs, FDA stated that its prior general enforcement discretion policy for LDTs did not apply to DTC tests,^132^ which it believes require a heightened level of oversight especially when they are ‘used by consumers to make potentially significant healthcare decisions without the involvement of a learned intermediary in a legitimate health care practitioner-patient relationship.’^133^ As a result, FDA categorized DTC tests into multiple subcategories with different regulatory pathways, with varying degrees of regulation.^134^ The subcategories include genetic carrier screening tests, genetic health risk tests, pharmacogenetic tests, cancer predisposition tests, low-risk general wellness tests, and ancestry tests.^135^

#### IV.A.4. Exemptions for General Wellness and Low-Risk Tests

Although the issue of how LDTs will be regulated remains uncertain in the longer term, if FDA is able to successfully issue regulations for DTC tests or software-based tests, of relevance for the regulatory pathway of DTC microbiome tests would be whether the test is used for general wellness purposes *and* is low risk. If so, assuming FDA is able to regulate DTC tests, under its current framework it would likely not require premarket review of the DTC microbiome tests as it views them as low-risk general wellness tests.^136^ This policy stems from the Agency’s Industry Guidance initially drafted in 2015, reissued in 2016^137^ and finalized in 2019,^138^ as well as the 21st Century Cures Act.^139^

FDA does not define ‘low risk tests’ but provides some guidance as to what *does not* constitute a ‘low risk’ general wellness product. For example, products that are invasive or implanted would not be low risk; nor would products that involve an ‘intervention or technology that may pose a risk to the safety of consumers and other persons if specific regulatory controls are not applied.’^140^ The Agency also provides a number of examples of products that would and would not be considered low risk.^141^ None of the examples in FDA’s Industry Guidance documents for ‘low risk’ tests include ‘health tests’ that might have false positives or false negatives that could lead to consumer harm.

For DTC tests intended for moderate- or high-risk medical purposes, FDA has, at least up until now, reviewed the tests for analytical validity, clinical validity, and the company’s claims about its product.^142^ Additionally, FDA has considered whether the instructions for collecting the specimen and interpreting the test results can be understood by the consumer without involvement of a physician.^143^ If FDA determined that a DTC test met each of these criteria, it would ‘clear’ the test for marketing. The earliest DTC genetic tests cleared by FDA were 23andMe’s carrier screening test for Bloom Syndrome in February 2015^144^ and its genetic health risk (GHR) tests^145^ in April 2017.^146^ FDA granted 23andMe’s requests for marketing the carrier test based on a 510(k)submission and, for its GHR tests, based on a *de novo* Class II medical device submission. The approvals came after a contentious history during which FDA issued warning letters in 2010 to 23andMe and other DTC genetic testing companies stating that their tests were medical devices and would be regulated as such. Despite the warning letters, 23andMe continued to market its tests. In 2013, FDA ordered 23andMe to stop marketing its DTC tests because the company had not established the tests’ analytical or clinical validity.^147^

For the GHR tests, the company was required to adopt general as well as numerous special controls. These special controls included specific information to consumers in labeling and test reports about the limits of the test results, specifically that other companies offering a genetic health risk test for the same disease may use different genetic variants and thus get different results; and that other factors, such as the environment or lifestyle risks, may play a role in whether the consumer develops the disease. FDA also required that certain information be provided to health care providers, such as a statement that the test is not intended to diagnose a disease and that any diagnostic decisions made should be based on other diagnostic testing and/or other information that the provider deems appropriate. Additionally, the company was required to ‘use a sample collection device that was FDA-cleared, -approved, or -classified as 510(k) exempt, with an indication for in vitro diagnostic use in over-the-counter DNA testing.’^148^ Finally, the FDA mandated extensive labeling requirements designed to help consumers understand and interpret the test results along with a hyperlink to the manufacturer’s website where the manufacturer was to make available all the special control information requirements.^149^

Subsequent to its approval of 23andMe’s GHR tests, FDA cleared several pharmaco-genetic tests and a cancer risk test developed by 23andMe.^150^ As of October 2024, 23andMe was the only company to have had its DTC tests cleared for marketing by FDA.^151^ Despite FDA asserting its authority over certain health-related genetic tests, many DTC genetic and microbiome testing companies likely assumed they were low-risk general wellness tests and, thus, not regulated by FDA as medical devices.

Although FDA had issued its draft Industry Guidance for low-risk wellness products in January 2015 and a subsequent draft in July 2016, Congress passed the 21st Century Cures Act in December 2016,^152^ exempting certain DTC tests using software from medical device regulation.^153^ The legislation listed five bases for exemption. Among the exemptions was that the software be ‘for maintaining or encouraging a healthy lifestyle and unrelated to the diagnosis, cure, mitigation, prevention, or treatment of a disease or condition.’^154^ The subsequent Industry Guidance by FDA^155^ clarified that the Agency did not intend to regulate software-based tests that are intended *only* for ‘general wellness’ as medical devices.^156^ The Agency defined a general wellness product as one that is low risk and has an

intended use that relates to maintaining or encouraging a general state of health or a healthy activity, or… that relates the role of healthy lifestyle with helping to reduce the risk or impact of certain chronic diseases or conditions and where it is well understood and accepted that healthy lifestyle choices may play an important role in health outcomes for the disease or condition.^157^

This definition breaks down products that meet the general wellness category into those that do not make any reference to a disease or condition and those that do.^158^ Included in the prior group are ‘claims to promote or maintain a healthy weight, encourage healthy eating, or assist with weight loss goals.’^159^ Not included in this group are claims ‘to restore a structure or function impaired due to a disease or condition.’^160^ The second group, which makes reference to diseases or conditions, is further divided into those ‘intended… to promote, track, and/or encourage choice(s), which, as part of a healthy lifestyle, may help to reduce the risk of certain chronic diseases or conditions… [or] help living well with certain chronic diseases or conditions.’^161^ Claims made by this second group must be based on references ‘where it is well understood that healthy lifestyle choices may reduce the risk or impact of a chronic disease or medical condition.’^162^ By ‘well understood,’ the association between the healthy lifestyle choices and health outcomes must be generally accepted in the scientific community.^163^

While the 21st Century Cures Act likely bolstered the view of software-based DTC microbiome testing companies that they were exempt from medical device regulation, this assumption may have been wrong. DTC microbiome-based tests may not meet the requirements for ‘general wellness.’ Claims made by many of the companies go beyond general wellness claims. As previously stated, claims that a product may ‘restore a structure or function impaired due to a disease or condition’ are not exempt general wellness claims.^164^ Many of the claims made by DTC microbiome testing companies link the tests to the ability to restore a structure or function of the gut or vaginal microbiome, eg the structure of the microbiome is impliedly and in some cases explicitly impaired (in ‘dysbiosis’) due to a disease or condition. While some companies reference specific diseases, such as IBS or bacterial vaginosis, others *imply* that such dysbiosis is due to an abnormal condition. These claims are being made even though there is no substantial evidence that microbiome test results can indicate these conditions or disorders and, even if they could, such associations are not ‘well understood’ or accepted in the scientific community. Moreover, there is no acceptance in the scientific community that choices to take certain supplements or eat certain foods will resolve dysbiosis, improve health, or prevent disease. A final argument that these tests do not meet the exemption for general wellness tests is that they appear to be marketed to individuals with a health problem or condition, not to healthy individuals.

#### IV.A.5. Exemptions from Regulation under the 21st Century Cures Act

If DTC microbiome-based tests were determined to be medical devices (ie not considered low risk or general wellness tests and not exempt as LDTs), the question would remain whether they would be exempt from device regulation under the other 21st Century Cures Act software exemptions.^165^

In addition to exemptions for software products that encourage or promote a healthy lifestyle, the 21st Century Cures Act exempts from medical device regulation four software functions.^166^ Of these, the most relevant for DTC microbiome-based tests is likely software that is intended for

transferring, storing, or displaying clinical laboratory test or other device data and results, findings by a health care professional with respect to such data and results, general information about such findings, and general background information about such laboratory test or other device unless such function is intended to interpret or analyze clinical laboratory test or other device data, results, and findings.^167^

How this provision applies to DTC microbiome-based testing requires an understanding of the different software functions associated with these tests. Most testing companies likely employ several software functions in the testing process. These may include (1) analysis of the composition of the sample, (2) a comparison of the sample composition with the ‘average’ sample composition from company data and/or with ‘healthy’ or ‘unhealthy’ populations, (3) flagging areas of concern (those that fall outside the ‘norm’ or are characteristic of unhealthy individuals), (4) displaying results, and (5) making recommendations based on test outcomes. As such, the tests would likely be considered multiple function device products.^168^ Arguably, functions 1 (analyzing the composition of the sample) and 5 (making recommendations) would involve the interpretation or analysis of device data and would be ‘device functions’ subject to premarket review. The other three functions more likely would be considered ‘other functions.’^169^ Under the Cures Act^170^ and FDA Industry Guidance, ‘FDA may assess the impact of “other functions” when assessing the safety and effectiveness of the device functions-under-review of a multiple function device product.’^171^ FDA Guidance further provides that the Agency will ask two questions when assessing a product with multiple functions: ‘(A) Is there an impact on the safety or effectiveness of the device function-under-review as a result of the “other function”?; and, if there is an impact, (B) Could the impact result in increased risk or have an adverse effect on performance of the device function-under-review, i.e., negative impact?’^172^

Based on the Guidance document, FDA would likely evaluate these functions holistically rather than evaluate each one separately, as the recommendation function is dependent for its accuracy on both the device functions (numbers 1 and 5) and the ‘other functions,’^173^ ie if the analysis of the sample is incorrect or the data regarding the ‘normal’ or ‘healthy’ person is inaccurate, that will affect the recommendations. Thus, under the provisions for transferring, storing, converting, or displaying data, these functions would likely not be exempt because at least the first and fifth functions fall under the transfer provision exception, ie the software ‘interpret[s] or analyze[s] clinical laboratory test or other device data, results, and findings.’^174^ Furthermore, even if the software is not considered a ‘clinical’ laboratory test, as required under the transferring provisions, because it is not aimed at physicians, if it is considered a device, it would be interpreting or analyzing ‘other device data, results and findings,’^175^ and would still not be exempt from regulation.

The other exemption under the 21st Century Cures Act that is of possible relevance is referred to as the clinical decision support (CDS) software exemption.^176^ It exempts software that is for

(i) displaying, analyzing, or printing medical information about a patient or other medical information…;
(ii) supporting or providing recommendations to a health care professional about prevention, diagnosis, or treatment of a disease or condition; and
(iii) enabling such health care professional to independently review the basis for such recommendations that such software presents so that it is not the intent that such health care professional relies primarily on any of such recommendations to make a clinical diagnosis or treatment decision regarding an individual patient.^177^

The software must meet all three tests to be exempt. These provisions limit FDA’s regulatory authority because clinical decision-making constitutes the practice of medicine and is governed largely by state medical practice regulations and tort law.^178^ It is likely that these provisions also reflect the assumption that, with a health care provider acting as an intermediary, consumers will be less likely to misinterpret a test result.

It is questionable whether these provisions would apply to DTC microbiome-based tests as they are marketed as tests for consumers and, for the most part, bypass the need for physician involvement. While this is generally the case, some physicians (eg functional medicine physicians) will order these tests for their patients and use them as part of their diagnostic process. However, they use them along with an array of other tests to build a diagnostic picture when the presenting symptoms are not easy to interpret.^179^ Also, as indicated *supra*, a few microbiome testing companies market directly to physicians.^180^ In these cases, the tests might meet elements (i), (ii), and (iii) of the exemption requirement.

Furthermore, the CDS exemption could apply if it covers dietitians or nutritionists as the exemption applies to ‘health care professionals,’ not just physicians. In its Industry Guidance, FDA states that it

uses the term health care professional to mean an individual who is licensed, registered, or certified by a State, territory, or other governing body, to administer health care, including but not limited to, nurse practitioner, registered nurse, licensed practical nurse, clinical social worker, dentist, occupational therapist, pharmacist, physical therapist, physician, physician assistant, psychologist, respiratory therapist, speech-language pathologist, technologist, or any other practitioner or allied health professional.^181^

The broad inclusionary language of ‘any other practitioner or allied health professional’ would seem to encompass these two professionals. Approximately half the states require licensure of dietitians and almost a dozen require licensure of nutritionists.^182^ Many DTC microbiome testing companies employ nutritionists or dietitians to consult with consumers about their test results and make recommendations regarding diet or dietary supplements allegedly based on those test results. Each state that licenses these practitioners has its own definition of the practice of dietetics and nutrition. An example from one state provides that the practice of dietetics includes, among other things, ‘applying scientific research to the role of food in the maintenance of health and treatment of disease.’^183^

Some DTC microbiome-based test software could meet the requirement that it is ‘supporting or providing recommendations to a health care professional about prevention, diagnosis, or treatment of a disease or condition,’ a requirement for CDS software.^184^ However, in the context of a physician, dietitian, or nutritionist, it would also have to ‘enable such health care professional to independently review the basis for such recommendations that such software presents so that it is not the intent that such health care professional rely primarily on any of such recommendations to make a clinical diagnosis or treatment decision regarding an individual patient.’^185^ Thus, it would depend on how much the provider is relying on the software recommendation versus providing their own assessment of the test results.^186^

To the extent that the tests do not meet the requirements for the other exemptions, ie are not considered general wellness tests, etc. *and* are being used by health care professionals, the CDS software exemption provisions would come into play. However, there is also an exception to the software exemption that may be relevant, ie if ‘the software function is intended to acquire, process or analyze a medical image or a signal from an in vitro diagnostic device or a pattern or signal from a signal acquisition system’ then it will be regulated as a medical device^187^ if it meets the medical device definition.^188^ The term *pattern* ‘refers to multiple, sequential or repeated measurements of a signal or from a signal acquisition system,’ such as next-generation sequencing (NGS).^189^ Specifically, ‘software functions that process or analyze the genetic sequence or patterns from an NGS analyzer to identify genetic variants or mutations or their clinical implications or relevance do not meet’^190^ the exemption criteria and would be regulated as a medical device. While clear this provision applies to DTC genetic testing software, there does not appear to be a reason why it would not also apply to DTC microbiome tests in which NGS is used to identify the microorganisms in a sample.

Thus, even if DTC microbiome-based testing software met requirements (i)–(iii) for the CDS exemption, it likely falls under the exception to the exemption because it is intended to ‘acquire, process, or analyze a medical image or a signal from an in vitro diagnostic device or a pattern or signal from a signal acquisition system’ and would not be exempt from the medical device regulations.^191^

### IV.B. Regulating Claims and Protecting Consumers from Misbranding and Unsubstantiated and Misleading Information

For tests that market directly to consumers, the most likely basis for exemption from FDA medical device regulation would be that the tests are low-risk general wellness tests.^192^ However, this is not a certainty and would depend on the claims companies are making and the potential harms that result from their recommendations. If consulted, FDA would consider whether such tests are marketed as non-medical, general wellness products before determining whether they would be exempt from premarket review.^193^ If the Agency were to determine that the tests are for general wellness, in addition to not exercising oversight of their analytical or clinical validity, FDA would not regulate the company’s marketing claims if those claims fit within the parameters described above, ie (1) they are ‘health claims’ and do not make any reference to diseases or conditions, or (2) they focus on a healthy lifestyle and make reference to diseases or conditions only if it is ‘well understood and accepted’ that a healthy lifestyle choice can reduce the risk of the chronic disease or condition that the product references.^194^ If FDA were to determine that the manufacturer’s intent is to market a product that diagnoses, treats, or reduces the risk of a disease or condition, despite the manufacturer’s claim that it is a general wellness product, FDA could decide that the product is misbranded and should be treated as a medical device.^195^

Under the FD&C Act, all medical devices, regardless of risk classification, are subject to general controls that relate to misbranding.^196^ Misbranding includes labeling that is false or misleading; does not state the name of the manufacturer, quantity, or adequate directions for use; or describes an intended use that is not approved by FDA.^197^ FDA also is tasked with ensuring that products are not misbranded by using false or misleading advertising.^198^ Although FDA jurisdiction over advertising is limited to restricted medical devices,^199^ it has jurisdiction over promotional labeling of both restricted and unrestricted medical devices.^200^ Some examples of promotional labeling are product brochures, print ads, and internet websites.^201^ Although, by regulation, promotional labeling refers to drug products, FDA has applied the concept to medical devices.^202^ FDA also assesses the risk and benefit claims for medical devices that are not low-risk general wellness products to ensure that the risk–benefit profile of a given product is balanced; if not, the overall promotional message (ie not the individual claim) is considered to be false or misleading.^203^

FDA has actively regulated some DTC genetic test marketing claims. For example, on April 4, 2019, FDA sent Inova Genomics Laboratory a warning letter for not obtaining marketing clearance or approval for its MediMap genetic tests.^204^ The Agency flagged Inova for its website claim that ‘the MediMap tests [are] genetic tests for predicting medication response’ and determined that the product is a diagnostic medical device for which clinical validity had not been established.^205^ FDA was concerned that providing test results directly to patients ‘could lead to patients inappropriately increasing, decreasing, or stopping their medication without their physician’s involvement, which poses significant risks to patient safety.’^206^ FDA also clarified that the MediMap LDT was not exempt from premarket approval because the agency retains its discretion to regulate LDTs that pose a risk to public health.^207^

In addition to the FD&C Act, the Federal Trade Commission (FTC) Act protects consumers from unfair, deceptive, and fraudulent business practices.^208^ Under Section 5 of the Act, advertising claims are deceptive if they mislead consumers in their conduct or decisions with respect to a product. Product advertising includes print and broadcast materials and internet advertisements.^209^ For unrestricted medical devices, FTC considers whether claims are ‘truthful, non-misleading and substantiated by “competent and reliable scientific evidence.”’^210^ In practice, FTC is less concerned about the truth or falsity of a claim than whether the claim is substantiated by reasonable supporting evidence.^211^ Furthermore, the substantiation must be material in that a consumer would be less likely to rely on the company’s claim if the company ‘did not have a reasonable basis for believing [the claim] to be true.’^212^

FTC, like FDA, has enforced its prohibition of false and misleading claims against genetic test manufacturers. In 2014, FTC brought an enforcement action against GeneLink, Inc. and a former subsidiary company because they claimed that their customized nutritional supplements based on the DNA analysis of a consumer-supplied cheek swab could treat diabetes, heart disease, arthritis, insomnia, and other ailments.^213^ GeneLink settled with FTC by agreeing not to make disease or health claims related to ‘genetic disadvantages’ unless ‘the claim is true and based on competent and reliable scientific evidence,’ ie supported by ‘at least two adequate and well-controlled studies.’^214^

Many of the DTC microbiome testing companies we reviewed make general wellness claims, including claims about how the tests’ results can help consumers improve their nutrition, weight, mood, energy, and/or fitness.^215^ These claims would likely exempt the tests from medical device regulation. However, numerous companies make healthy lifestyle claims about (1) identifying and reducing disease risks for intestinal complaints or vaginal issues, or (2) improving living with a chronic disease.^216^ These latter claims arguably do not meet the criteria for exemption because claims that the tests can identify disease risks or the supplements the companies sell can improve the microbiome and ultimately reduce risks of disease are claims that are not generally accepted by the scientific community. For example, the following statements made on the promotional materials or websites of DTC microbiome testing companies may fall short of meeting the requirements for healthy lifestyle claims:

1. *[Our] personalized probiotics support GI motility, SIBO, leaky gut, and overall digestive wellness.*
2. *[Our test results] can reveal important information about the root cause of many common gastrointestinal symptoms and non-GI conditions including:*
  - *Indigestion/reflux*
  - *Abdominal pain/cramps*
  - *Diarrhea*
  - *Constipation*
  - *Inflammatory bowel disease (IBD)*
  - *Irritable bowel syndrome (IBS)*
  - *Atopic dermatitis/eczema*
  - *Allergies*
  - *Autoimmune diseases*
  - *Mood disorders (depression)*
  - *Joint aches*
  - *Diabetes*
  - *Weight issues*
3. *This test is useful for women who have or are at risk of pregnancy and fertility issues, bacterial vaginosis, candida, pelvic inflammatory disorder and other vaginal problems.*
4. *[Our test can help]*
  - *Improve fertility and prenatal health to support full-term pregnancy*
  - *Reduce the risk of cervical cancer and endometriosis*
  - *Prevent recurring urinary tract infections (UTIs), bacterial vaginosis (BV), and aerobic vaginitis*
  - *Decrease risks of sexually transmitted infections (STIs) and human papillomavirus infection (HPV)* ^217^

Additionally, even if the company does not mention a specific disease or condition, if a consumer understands the claim to be referring to a disease or condition, FDA may consider it a more than low-risk product.^218^ Also of possible relevance, under regulations for dietary supplements, a claim that a product ‘has an effect on an abnormal condition associated with a natural state or process, if the abnormal condition is uncommon or can cause significant or permanent harm,’ would be a disease claim.^219^

While a claim regarding a probiotic may be permissible under the Dietary Supplement Health and Education Act of 1994 (DSHEA)^220^ as a structure/function claim,^221^ the Act does not apply to claims about medical devices. However, in its Guidance about low-risk general health and wellness devices, FDA has stated that structure function claims that do not refer to a disease or condition would fall under the low-risk general health and wellness exemption, whereas claims ‘to restore a structure or function impaired due to a disease or condition’ would fall outside the general wellness exemption.^222^ If the claims are not considered to fall under the exemption requirements, FDA could, among other things, issue a warning letter to the manufacturer that it is operating in violation of the FD&C Act. As stated above, the Agency will also consider whether a consumer would think the claim refers to a disease and, if so, will categorize the claim as a disease claim.^223^

### IV.C. Other Potential Legal and Regulatory Issues Raised by DTC Microbiome-Based Tests

While safety, accuracy, and truthfulness are perhaps the main concerns of regulators, consumers have also become concerned about privacy, especially as it relates to their health information, and how their data may be used by companies with whom they do business. In the context of genetic data collection, consumers have alleged that companies are using the data they collect for research without adequate consent, and that law enforcement may have access to genetic databases for forensic purposes.

#### IV.C.1. Protection of Consumer Data

When FDA approved 23andMe’s tests for predicting the genetic risk of disease, the Agency did not address the potential for invasion of privacy in the case of a data breach or company use of the genetic data for purposes unapproved by the consumer. A significant part of 23andMe’s business model, however, was the collection of genetic data from consumers to sell to third parties including researchers, insurers, and pharmaceutical companies.^224^ Anticipating a lucrative return on investment from these sales, 23andMe subsidized the cost of its genetic services to consumers and, thereby, collected even more data to sell to third parties.^225^ We assume DTC microbiome testing companies are also selling consumer data to third parties.^226^

In the USA, there is limited law protecting collection of patient data by commercial entities. In the European Union (EU), in contrast, protection of consumer data is governed by the General Data Protection Regulation (GDPR), which establishes how personal data (including genetic data) may be processed and stored by companies doing business in the EU.^227^ In the USA, however, there is no overarching federal data protection law.^228^ Rather, the US digital marketplace is addressed by bifurcated law^229^ that covers governmental entities *or* private entities^230^ and sectoral laws that apply to entities based on the type of information they collect and use.^231^ An example of a sectoral law is the Health Insurance Portability and Accountability Act (HIPAA),^232^ which applies only to health data collected by covered health care entities.

HIPAA protects the privacy and security of a patient’s individually identifiable information (eg name, address, birth date, and Social Security number) and applies to health care industry entities and certain of their business associates.^233^ HIPAA also applies to sensitive health information such as genetic data, but it does not apply to deidentified genetic data that is collected by DTC genetic testing companies not affiliated with the health care industry.^234^ Since DTC microbiome testing companies are not health care providers, HIPAA does not apply to them.^235^ This creates a regulatory gap that allows companies to avoid federal oversight of genetic data privacy. In fact, if they perform metagenomic sequencing, they generate human genetic information (especially in vaginal or buccal samples). That information could be useful to the company, and its use is not regulated.

DTC genetic testing companies exploit this regulatory gap through the use of privacy policies and terms of service (ToS) agreements that enumerate how a company collects and uses consumers’ genetic data.^236^ As a condition of using a company’s services, consumers must consent to the contents of the privacy policy and ToS, but the complexity (ie unintelligible legalese), ambiguity, and lack of standardization make this form of consent largely ineffective.^237^ Furthermore, many companies ‘reserve the right to unilaterally change their ToS, often without users’ consent.’^238^ After a consumer consents to the terms, a company may post changes directly to the ToS and advise the consumer to check the terms periodically to become aware of them. If a consumer continues to use the company’s services after the ToS have changed, the consumer has impliedly accepted the change(s). Although such ‘bait and switch’ tactics that unilaterally make material changes to the ToS without notice to consumers may constitute a cause for legal action, most consumers do not have the resources to assert a legal challenge and are faced with either accepting the new terms or discontinuing their use of the company’s services.^239^

In practice, most consumers consent without reading the privacy policy or ToS and, therefore, do not understand the potential uses of their data to which they are asked to consent.^240^ Furthermore, notwithstanding FDA’s approval of GHR tests as a type of medical device, DTC genetic tests are considered commercial transactions and not medical tests for the purposes of patient informed consent.^241^ And, although most states have some form of statute protecting the privacy of genetic data,^242^ the scope of these laws vary widely, leaving the USA with no comprehensive privacy protection for genetic data.

Other laws, however, may provide consumers with some protection. In the case of GeneLink, in addition to bringing false claims charges regarding its customized nutritional supplements, FTC charged the company with ‘deceptively and unfairly’ stating that it had taken ‘reasonable and appropriate security measures to safeguard and maintain personal information collected from nearly 30,000 consumers.’^243^ FTC asserted that the company failed to protect consumers’ personal information including their genetic information.^244^ Thus, under the FTC Act, not only can companies not make false claims about their products, they also may not make false claims about their privacy policies.

DTC microbiome testing companies, like their genetic testing counterparts, use privacy policies and ToS to obtain consent for the collection and use of consumer data. Consumers impliedly consent to the collection and use of their data when they click through the privacy policy and ToS to register to use the company’s services. Each of the US DTC microbiome testing companies we reviewed clearly state that failure to consent to their privacy policy or ToS results in reduced (or zero) use of the company’s services.

The 23 privacy policies we reviewed were generally easy to understand and most explained in detail how they collect, use, and share consumer data. Several policies also claimed to adhere to privacy laws such as GDPR and state laws that apply to consumers in those jurisdictions. Just like genetic testing companies, DTC microbiome testing companies that do not analyze samples supplied by a physician’s office are not covered entities under HIPAA and, even if they were, they are not bound by HIPAA requirements if they anonymize consumers’ sensitive (ie health) data before it is used or shared.

#### IV.C.2. Use of Consumer Data for Secondary Research

When DTC microbiome testing companies use privacy policies to obtain consent to collect consumer data, they also rely on these policies to inform consumers about how their data might be used. For example, their data might be used in secondary research, eg attempting to find a link between microbiome composition and a specific disease. The Federal Policy for the Protection of Human Subjects (ie Common Rule) establishes an overarching framework for the regulation of federally funded human subjects research or research conducted at an academic research institution that receives federal funds.^245^ Although the Common Rule does not cover privately funded research conducted by non-academic entities, clinical trials conducted as part of premarket approval for medical devices are subject to FDA regulations governing the protection of human subjects. These provisions, which are similar to the Common Rule guidelines, have the effect of extending the Common Rule to private entities seeking FDA premarket approval for a drug or device.^246^ Since, however, the DTC microbiome-based tests described herein are not being regulated as medical devices, such rules would not apply to secondary research conducted with the data obtained from these tests.

Although not legally required for these tests, informed consent ‘is essential to the conduct of ethical research.’^247^ The requirement for informed consent was crafted at a time when human subjects research was conducted within the confines of randomized controlled trials that utilized individually identifiable information.^248^ In the case of DTC genetic tests and microbiome tests, however, data is collected when the consumer consents to use the service and can be anonymized and aggregated for ‘undefined future research purposes.’^249^ Most of the 23 US DTC microbiome testing companies we reviewed for this article obtain consent through their privacy policies to use consumer data for research or other unspecified commercial and non-commercial purposes, which could include secondary research. Half of the reviewed companies using consumer data to conduct secondary research indicate on their websites that they aggregate or otherwise deidentify the data before it is used.

#### IV.C.3. Discrimination Based on DTC Microbiome Test Results

With the absence of federal privacy protections for genetic data comes the interrelated risk of discriminatory use of this data. Unfettered access to consumers’ genetic data gives employers and health insurers an unfair advantage in making employment and insurance coverage decisions. As a result, Congress passed the Genetic Information Nondiscrimination Act (GINA) in 2008.^250^ Proponents of the bill argued that federal protections against genetic discrimination were needed to advance personalized medicine by making consumers willing to participate in genetic research and comfortable with having a genetic test for clinical purposes.^251^ Critics of the bill argued that the legislation would not resolve state law inconsistencies and would increase frivolous litigation, but, to date, GINA has not been heavily litigated.^252^

GINA prohibits health insurers from requesting or requiring a prospective or currently insured person from undergoing a genetic test or using their genetic information to make eligibility determinations or decisions regarding coverage, underwriting, or premium-setting.^253^ Additionally, GINA prevents employers from using a person’s genetic information in hiring, firing, promotion, pay, or job assignment decisions, and employers cannot request, require, or purchase genetic information about an employee.^254^ Forty-nine states also have statutory restrictions on the use of genetic information or genetic testing by health insurers, and 35 states extend these restrictions to employers.^255^ Twenty-four states also limit the use of genetic information by life, disability, and long-term care insurers.^256^

The application of GINA to DTC microbiome-based tests requires that these tests fit within the definition of genetic information or genetic tests. Under GINA, genetic information includes genetic test results. A genetic test result is defined as ‘an analysis of human DNA, RNA, chromosomes, proteins, or metabolites, if the analysis detects genotypes, mutations, or chromosomal changes.’^257^ If an employer requests an employee’s microbiome test results, the application of GINA would be based, first, on whether the test results are considered ‘human’ DNA. Whether microbiome DNA is human DNA has not been definitively decided by any court, government agency, or scientific body. In fact, there is some debate within the regulatory and scientific community about whether microbiome material obtained from inside or on the human body is human since it is made up of non-human microorganisms.^258^ However, while microbiome samples are primarily made up of microorganisms, they may also include some human tissue.^259^ The second issue is whether, as a practical matter, microbiome tests could be used by an employer or insurer to discriminate against the employee or insured. For example, failure to regulate these tests as SaMD could result in the industry developing algorithms with inherent biases or software used to identify individuals exposed to certain drugs or toxins or living in certain neighborhoods.^260^ Given these regulatory gaps, consumers may be at risk of insurance or employment discrimination.

Consumers of DTC microbiome test results may also be at risk of other kinds of discrimination or stigma. Many testing companies collect personal information about a consumer’s lifestyle that may include diet, drug use, and sexual practices. Women with specific vaginal microbiome profiles may be labeled at risk for bacterial vaginosis, fertility problems, or premature births. Such information, if inappropriately shared, could target them for drug promotions or advertisements that are embarrassing or stigmatizing. While laws against discrimination may not address this type of stigma, laws aimed at prohibitions on companies selling this information to other companies without consumer consent may be a way to curtail such practices.

#### IV.C.4. Use of DTC Microbiome Test Results for Law Enforcement Purposes

The use of genealogy databases such as 23andMe, Ancestry.com, and GEDmatch by law enforcement for criminal investigations^261^ raises the question of whether a law enforcement agency could obtain a consumer’s microbiome test results for forensic purposes. Recent research suggests that each individual has a personal microbiome that can be used to identify them.^262^ This so-called ‘microbial cloud’^263^ may one day be used to reveal aspects of, and predict, a person’s habits and lifestyle.^264^ For example, ‘neighborhood built environment characteristics, such as increased interaction among home occupants and indoor ventilation, may help determine the presence of microbiome species’^265^ that could identify an individual from their microbiota and possibly place them at a specific location.

What is deeply troubling to many in the genealogy community is that this area is wholly unregulated. Health law scholars who have written about this issue in the context of genetic information argue that it implicates Fourth Amendment privacy rights.^266^ The Fourth Amendment of the U.S. Constitution guarantees ‘the right of the people to be secure in their persons, houses, papers, and effects, against unreasonable searches and seizures….’^267^ Under this constitutional right, the U.S. Supreme Court identified several individual privacy rights that the government is obligated to protect. If microbiome-based information could provide law enforcement with meaningful evidence of identity in the commission of a crime that could not otherwise be obtained, it would raise Fourth Amendment privacy concerns. To the extent that an individual can be identified by their microbiome, use of that information by law enforcement could put an individual at the scene of a crime. Additionally, an individual’s gut microbiome analysis could indicate that they used illegal drugs or that they visited a foreign country where the USA prohibits its citizens from travel.

## V. RECOMMENDATIONS

Regulation of the DTC microbiome testing industry, like the DTC genetic testing industry, requires a regulatory framework that will address the health and safety risks associated with these products. Such risks may include consumers improperly self-medicating, avoiding care from a provider, or taking supplements instead of their physician-prescribed drugs.

Furthermore, many of the companies make claims that mislead consumers into believing that there is a proven connection between their microbiome composition and their health or specific diseases or health conditions, that changing their diet or taking recommended supplements can change their microbiome, and that changing their microbiome can improve their health. Many individuals who use these tests are ill and suffer from chronic conditions that significantly affect their quality of life. When ‘traditional’ therapies for their conditions are not effective or do not lead to full resolution of their symptoms, they seek out other information and hold out hope for a treatment that will provide relief from their pain and other debilitating symptoms. Physicians have reported seeing patients who have used DTC microbiome tests, coming to them after following harmful dietary restrictions or undergoing fecal microbiota transplants without first consulting a medical expert.

Consumers may also not be aware that their test results are being sold by DTC microbiome testing companies, and that there is absolutely no privacy protection of their data. As a result, they could be required to disclose test results to insurers or employers who may use the information to deny them health insurance or employment. Finally, to the extent that one’s microbiome test results can be used to identify them, law enforcement may be able to obtain such results from companies and use them for forensic purposes.

Over a decade ago, the U.S. Government Accountability Office observed that DTC genetic tests were unreliable because different companies were reporting different results using the same samples.^268^ Similarly, a study conducted by NIST found that DTC microbiome testing companies that received the same samples reported different results.^269^ Like the early DTC genetic tests, the DTC microbiome testing companies are ‘nowhere near… being able to interpret’^270^ their test results. Both the analytical and clinical validity of the tests are highly suspect.

The manufacturers of the early DTC genetic tests led consumers to believe that the test result was linked to health and disease when in many cases it was not. The DTC microbiome testing companies are using the same playbook—leading consumers to believe the tests can inform them about their health and medical conditions when there is no substantiation of the relationship between test results indicating dysbiosis and risk of disease. While the DTC microbiome testing companies may not always name a specific disease, they want the consumer to believe that the test can diagnose a disease or health condition and the related dietary change or supplement they recommend can treat, mitigate symptoms, cure, or reduce the risk of a disease or condition. To be diagnostic or predictive, DTC microbiome-based tests would have to overcome their lack of analytical and clinical validation by developing consistent and curated reference databases and software algorithms to ‘ensure accurate, replicable, and comparable results.’^271^

Some may argue that the harms associated with false-positive or false-negative GHR tests are greater than those associated with DTC microbiome tests. In the case of the GHR tests, using the example of tests for BRCA1, a false-positive test could lead a consumer to experience anxiety or depression, or to have a preventive mastectomy or oophorectomy, which are invasive and serious procedures. A false-negative result could lead to a delay in seeing a physician or scheduling a mammogram that would risk not identifying breast cancer at an early stage. Risks associated with inaccurate microbiome test results can have similar harmful effects. A false positive could lead someone with gut symptoms to experience anxiety or to change their diet or take supplements in ways that are unnecessary or harmful. And, like the case of breast cancer, a false negative could lead to a delay in seeking treatment from a physician.

These harms and risks are largely a result of gaps in the regulation of the manufacture, marketing, and use of these tests. Under CLIA, the laboratories that analyze these tests are inadequately regulated because, even if they are CLIA certified, the certification process does not ensure analytical validity. A major shortcoming of the CLIA regulatory process for DTC microbiome tests is that there are no established proficiency tests for them. Moreover, although FDA attempted to promulgate regulations that would roll back its enforcement discretion policy for LDTs in 2024, those regulations were recently vacated by a federal district court.^272^ This court decision undermines FDA’s ability to regulate this entire category of tests, ie LDTs, at least in the short term. However, assuming FDA remains able to regulate DTC tests, DTC microbiome testing companies appear to have assumed that these tests are low-risk devices for general wellness purposes and thus exempt from medical device regulation.

Many of these tests do not satisfy the requirements for exemption from medical device regulation as specified by the *American Clinical Laboratory Association v. FDA* case or under the 21st Century Cures Act and the FDA’s own guidance documents—they do not meet the definition of an LDT as described by the Court and are not low risk; nor do they meet the exemptions for software designed to improve health or encourage a healthy lifestyle; and they do not meet the exemptions for transferring and displaying test results or for clinical decision-making support.

To remedy these shortcomings, we advocate several regulatory reforms. First, we recommend that CMS, under CLIA, require DTC microbiome testing companies to report their testing methods and the algorithms they are using to measure the analytical validity of their tests. NIST is in the process of developing a reference standard for stool that can be used as a touchstone for the DTC microbiome testing industry.^273^ The reference material could significantly advance the field of microbiome-based testing.^274^ Once NIST makes available the reference standard, we advocate that as part of CLIA certification, CMS require DTC microbiome testing companies to use the standard material to evaluate the accuracy of their own procedures and as a source for comparison of consumer samples.^275^ In the meantime, DTC microbiome testing companies should be required to use a ‘lockdown method’ that helps ensure internally consistent test results by utilizing an analytical algorithm and reference database that does not change until the accumulation of new data necessitates the development of a ‘version 2.0’ of these tools.

Second, although we maintain that DTC tests do not meet the definition of LDTs put forth in the recent federal district court decision, we recommend that Congress pass legislation giving FDA clear authority to regulate LDTs either as medical devices or as a separate category.

Third, we think FDA should treat DTC microbiome tests in the way it treated the GHR tests marketed by 23andMe. The two types of tests are similar. The risks identified by the FDA with respect to 23andMe’s personal Genome Services—risk of incorrect understanding of the device and test system, incorrect test results, and incorrect interpretation of the test results—apply equally well to DTC microbiome-based tests.

As such, FDA should require DTC microbiome tests to submit an application similar to a *de novo* Class II medical device application for approval and to adopt general as well as several special controls. Like the requirements for 23andMe, the special controls should include information to consumers (in labeling and on websites) indicating

- the limits of the accuracy of the test results;
- that test results may be affected by external variables like low sample quantity or issues with sample collection, shipping, storage, or collection buffers;
- there is insufficient evidence to link the gut or vaginal microbiome to any disease or harmful condition or a predisposition to any disease or condition;
- the test may not enumerate all the microorganisms in the consumer’s gut or vagina and the microbiome may change over time;
- the detection of a pathogen does not necessarily indicate disease, and failure to detect a pathogen does not mean that the pathogen is not present;
- other companies offering DTC microbiome tests may follow different procedures and, thus, report different results;
- the test is not intended to diagnose diseases or health conditions or to be used in medical decision-making; and
- the test is not a substitute for a visit to a doctor or other health care professional.

Additionally, the FDA required 23andMe to meet labeling requirements ‘designed to help consumers understand and interpret the test results along with a “hyperlink” to the manufacturer’s… website where the manufacturer was to make publicly available all the special control information requirements.’ This should also be required for DTC microbiome test manufacturers. Furthermore, as it did with 23and Me, FDA should require the companies to use an ‘FDA-cleared, -approved or -classified as 510(k) exempt sample collection device.’^276^

Fourth, FDA and FTC should collaborate to ensure that companies making false or unsubstantiated claims receive warning letters and, if they persist in their actions, are charged with violation of federal law. FTC may be in the better position to bring such legal action, and its prior action against GeneLink may provide a template for regulatory enforcement against several of the DTC microbiome testing companies.^277^

Finally, in order to address regulatory gaps in HIPAA, GINA, and laws protecting human subjects, we advocate that the safeguards other scholars have recommended for DTC genetic testing companies, such as 23andMe, also be applied to DTC microbiome testing companies. These include requiring DTC microbiome testing companies ‘to obtain express consent to disclose [microbiome test results] with strict civil and criminal penalties if they do not,… limiting permissible disclosures [of microbiome test results] to research companies so that insurers, employers, and other third parties cannot exploit genetic information’; allowing consumers to have their genetic information ‘destroyed or deleted’; and providing consumers with ‘explicit information from DTC testing companies about how their DNA is being used,’ whether it be the specifics of the research or for marketing purposes.^278^

## VI. CONCLUSION

In sum, the DTC microbiome testing industry is growing both domestically and internationally. Consumers are paying hundreds of dollars to know if they have a healthy gut or vaginal microbiome. In some cases, these consumers are healthy and simply curious about their microbiome, but, in others, consumers have chronic illnesses, such as IBS or bacterial vaginosis, and are looking for information that may help them address their conditions. Consumers are relying on microbiome-based test results to purchase dietary supplements, including probiotics and prebiotics, and/or are changing their diet based on company recommendations. Yet, there are no standards for confirming the analytical or clinical validity of these tests, and company marketing claims border on disease claims. Yet, DTC microbiome testing companies are largely being ignored by the federal agencies that have the authority to regulate them, either because they lack the resources or they view them as exempt from medical device regulation under existing law or as a low priority.

We reviewed the regulatory frameworks that may apply to the current DTC microbiome testing industry and identified a number of potential regulatory gaps. While we conclude that the current regulations for DTC genetic tests likely apply to DTC microbiome-based tests, more effort needs to go into regulating the tests’ analytical validity by CLIA and regulating the tests’ analytical and clinical validity by FDA. The latter will require reassertion by FDA that it has the authority to, at a minimum, regulate DTC tests or Congressional action to clarify the law. Regulators should also reassess the decision that these tests are truly ‘low risk.’ Actual risks of the tests include providing consumers with recommendations that may be harmful to their health, or, at a minimum, are ineffective and a waste of money. Furthermore, it is unlikely that a number of the tests meet the definition of general wellness tests that have been exempt from medical device regulation. Finally, there are significant regulatory gaps in data privacy, human subject protections, and the use of consumer data for discriminatory or forensic purposes. The DTC microbiome testing industry has the potential to improve consumers’ lives, but these companies are not doing the research necessary to analytically and clinically validate their tests. Thus, there is a need for regulation that considers consumers’ safety from physical harms, unsubstantiated claims and false information, as well as economic and dignitary harms.

## Footnotes

1 In this article, we use the term ‘testing’ although a more appropriate term to describe the services provided by these direct-to-consumer companies might be ‘profiling’ or ‘analyzing.’

2 Kitty Knowles, *Are microbiome gut health tests “bullshit”?*, Sifted (Aug. 28, 2019), https://sifted.eu/articles/do-microbiome-tests-work.

3 The evolution of DTC genetic tests both influenced and posed challenges to the federal regulatory framework for DTC tests. The first DTC genetic testing companies emerged in the early 2000s, and 23andMe, the industry leader, launched in 2007. Megan A. Allyse et al., *Direct-to-Consumer Testing 2.0: Emerging Models of Direct-to-Consumer Genetic Testing*, 93 Mayo Clinic Proc. 113, 113 (2018). Although the genetic profiling services offered by these companies focused on non-medical ‘infotainment,’ such as ancestry tracing, the earliest genetic panels also included risk of disease information. *Id*. at 114*.*

4 Michael A. Conlon & Anthony R. Bird, *The Impact of Diet and Lifestyle on Gut Microbiota and Human Health*, 7 Nutrients 17 (2014), https://pmc.ncbi.nlm.nih.gov/articles/PMC4303825/.

5
*Evvy Vaginal Health Test*, Evvy, https://www.evvy.com/vaginal-microbiome-test (last visited July 4, 2025).

6
*Oral Health Intelligence Test*, Viome, https://www.viome.com/products/oral-health-intelligence (last visited July 4, 2025).

7
Parallel Health, https://www.parallelhealth.io/ (last visited July 4, 2025).

8
*See infra* Section II.A. *See also* Nadia Ayala-Lopez & James H. Nichols, *Benefits and Risks of Direct-to-Consumer Testing*, 144 Arch. Path. & Lab. Med. 1193, 1194–95 (2020).

9 For example, makers of the ‘DNA Warrior Gene Test’ state the test can tell whether you have a specific variant in the MAOA gene, ‘often referred to as the “warrior gene,” which has been linked to impulsive aggression as well as strong leadership and resilience.’ DNA Family Check, https://www.dnafamilycheck.com/product/dna-warrior-gene-test/?srsltid=AfmBOooePEDoHasPC4z98qWIKPO1IBe_sf6ujnh5iZBEbi0O1xJ3fVKDM7A&com_cvv=8fb3d522dc163aeadb66e08cd7450cbbdddc64c6cf2e8891f6d48747c6d56d2c (last visited July 4, 2025). The Cognitive Function DNA test claims it can help consumers ‘find potential causes of [their] brain fog, lack of mental clarity, or other cognitive issues; [b]ecome more intelligent by enhancing [their] genes.’ *Cognitive Function DNA Report*, Sequencing, https://sequencing.com/marketplace/genetic-test-for-iq-cognitive-function?srsltid=AfmBOoru-W5yBQH6fOrufq8WG-tEEY2aLaBCsC_OQaNrB0lZK9DkP3DF-F8 (last visited July 4, 2025).

10 Ayala-Lopez & Nichols, *supra* note 8, at 1195–97. As consumer interest in personalized medicine grew, the medical community warned that genetic testing companies were selling products with limited clinical utility; were ill-equipped to protect the privacy of consumer genetic data; and, without medical oversight, consumers would misinterpret their results and make poor health decisions.

11 U.S. Gov’t Accountability Office, *Direct-to-Consumer Genetic Tests: Misleading Test Results are Further Complicated by Deceptive Marketing and other Questionable Practices* 4–19 (2010) [hereinafter GAO Report].

12 Genetic Information Nondiscrimination Act of 2008, 42 U.S.C. § 2000ff.

13 David A. Simon et al., *Skating the line between general wellness products and regulated devices: strategies and implications*, 9 J. L. Bioscience 1, 1 (2022) (examining similar concerns for digital apps); *See also* David A. Simon et al., *At-home Diagnostics and Diagnostic Excellence: Devices vs General Wellness Products*, 327 JAMA 523, 523 (2022).

14
*Am. Clin. Lab. Ass’n v. U.S. Food & Drug Admin.*, Civil No. 4:24-CV-479-SDJ, 2025 WL 964238 (E.D. Tex. Mar. 31, 2025).

15
*See infra* Section III.C.

16
*See infra* Section IV.

17
*See infra* Section IV.A.2.

18
*See infra* Section IV.

19
*Direct to Consumer Microbiome Analyzing Market Expected to Grow at 11.8% by 2032*, Acute Mkt. Reports (2023), https://www.acutemarketreports.com/report/direct-to-consumer-microbiome-analyzing-market.

20 NIH Integrated Hum. Microbiome Project, https://commonfund.nih.gov/human-microbiome-project-hmp (last visited Sept. 16, 2024).

21 Researchers used 16S rRNA sequencing ‘to characterize the complexity of microbial communities at each body site.’ *Id.*

22 Eric Green, *The Human Microbiome Project Reaches Completion*, Nat’l Hum. Genome Rsch. Inst. (June 6, 2019), https://www.genome.gov/about-nhgri/Director/genomics-landscape/june-6-2019-Human-Microbiome_Project.

23 Using the Google search engine, we limited our internet search to English-language websites.

24 To identify DTC Microbiome Testing Companies, we conducted internet searches of these targeted phrases: “DTC Microbiome Testing”; “gut microbiome testing”; “buy gut microbiome testing”; “buy gut health test”; “vaginal microbiome testing”; “buy vaginal microbiome testing”; “buy vaginal health test.” These searches were conducted on multiple search engines periodically throughout a 3-year (2021–2024) period. During each search, we considered both the “Results” tab and any advertisements that were promoted based on the search. During each search, we considered the first 50–75 results. Companies that were entirely based outside of the USA, did not currently sell in the USA (based on their Terms of Service), and did not state an intention of expanding to the US market on their website were omitted from any analysis of CLIA certification but kept in the overall data set. Once a company was identified, we relied on its website and self-reported information to determine whether it markets itself as ‘direct-to-consumer.’ *DTC Microbiome Testing Companies* (as of November 1, 2024) U. Md. Carey L. Sch., https://www.law.umaryland.edu/academics/programs--centers/law--health-care-program/dtc-microbiome-grant/ (last visited July 4, 2025).

25 A few companies that came up in our search, however, do require a physician order to provide the test to a consumer, eg Diagnostic Solutions Laboratory requires a health care provider to order the test, but the company offers to help the consumer find a practitioner. GI-MAP – GI Microbial Assay Plus, Diagnostic Sol. Lab., https://www.diagnosticsolutionslab.com/tests/gi-map (last visited July 4, 2025) [hereinafter *Diagnostic Sol. Lab.*]. Some companies have recently moved to this model, eg as of Nov. 3, 2024, Microba’s website states that customers may only order their gut test through a physician. Microba, https://insight.microba.com/ (last visited July 4, 2025).

26 ‘The average stool test costs under $200 but may be more expensive depending on whether the consumer selects more in-depth kits that require other samples, such as blood or saliva.’ *See* Kristeen Cherney, *What’s Possible from Microbiome Testing at Home?*, Healthline (Oct. 29, 2021), https://www.healthline.com/health/microbiome-testing; *See also* Daniel Imperiale, *Microbiome Testing: Finding the Best Test,*  Innerbody (Dec. 30, 2024) https://www.innerbody.com/home-health-tests/microbiome-testing (listing prices from five companies). Test costs may also reflect the type of sequencing analysis performed by the company. Metagenomic sequencing is more costly and time consuming than 16S rRNA gene amplicon sequencing but provides more information about the species composition and metabolic functions encoded in the microbiome. *Id.*

27
*See eg*  Juno.Bio, https://www.juno.bio/ (last visited July 4, 2025) (stating that they offer a ‘1:1 Consultation’ in which ‘a vaginal coach will take you through your results.’). Although it is unclear what training a vaginal health coach has gone through, at least one company states that its coaches are ‘certified’ and provides brief descriptions of the backgrounds of each of their coaches on its website. Evvy, *Meet Evvy’s Vaginal Health Coaches*, https://www.evvy.com/blog/evvy-vaginal-health-coaches.

28
*See*, *Subscription-Based Business Models for Direct-to-Consumer Testing*, Zymo Rsch., https://files.zymoresearch.com/pdf/ds5045_subscription-based_business_models_for_direct-to-consumer_testing.pdf (last visited July 4, 2025). Viome and Ombre are among those companies offering subscription models. The Viome subscription plan, for example, provides monthly supplies of customized probiotics and prebiotics based on the results of the customer’s tests. Along with these products, the customer receives a free DTC test once a year. Daniel Imperiale, *Viome Reviews: Should you try a Viome gut microbiome test?*, Innerbody (Dec. 31, 2024), https://www.innerbody.com/viome-review#testing-with-viome. The vaginal testing companies similarly offer memberships. For example, Evvy offers a single vaginal microbiome test for $129, while ‘Evvy’s annual membership is $99 per test, shipped four times yearly, every three months.’ *Evvy vs. Juno*, Evvy (Feb. 18, 2025), https://www.evvy.com/blog/evvy-vs-juno.

29 Morgan G.I. Langille, *Exploring Linkages Between Taxonomic and Functional Profiles of the Human Microbiome*, 3 mSystems (2018).

30
*See eg* Kate M. Bermingham et al., *Effects of a personalized nutrition program on cardiometabolic health: a randomized controlled trial*, 30 Nat. Med. 1888 (2024).

31 These users can be their own customers, or companies may purchase data from health care providers or other researchers. For instance, one company claims that its ‘knowledge database’ contains data from over 7900 ‘studies’ conducted worldwide. *About Us*, Biomes, https://biomes.world/en/about-us/ (last visited July 4, 2025). For companies that use their own customers as the source of reference data, if they do not identify their reference group, customers who are tested at different intervals over time may, even if their microbiome does not change, see their results change.

32 Atlas Biomed sends its samples to a laboratory that uses genetic sequencing technology ‘to assess how “similar” your gut is to patients with obesity, type II diabetes, Crohn’s disease, ulcerative colitis and cardiovascular disease.’ Knowles, *supra* note 2.

33
*See eg BIOHM Gut Test*, BIOHM, https://www.biohmhealth.com/products/gut-report-kit-new (last visited July 4, 2025); *smartGUT™ Microbiome Test*, smartDNA https://www.smartdna.com.au/smartgut-microbiome-test/ (last visited July 4, 2025).

34
*Sample Report*, Thryve (Aug. 2, 2019), https://static.thryveinside.com/pdfs/thryve-sample-report.pdf. Thryve is now Ombre, which does not provide a similar report on its website, but see BiomeFX for an example of a current sample report. *Functional Microbiome Analysis*, BiomeFX (Aug. 23, 2021), https://static1.squarespace.com/static/5e8cc993f9169c7045248796/t/613f99e4368c6b72d5593fc8/1631558118019/BiomeFx3.5SampleReport.pdf; *see also Gut Report*, BIOHM, https://cdn.shopify.com/s/files/1/1713/4291/files/BIOHM_GutReport.pdf?7686022114233044609 (last visited Jan. 25, 2025) (providing a sample microbiome report).

35
Viome, *Demo Two’s Recommendations* 6 (2019), https://assets.ctfassets.net/qk4l4jfatr3e/5LmbY0DgNjXgFQ9kq8LWxa/f60f6d2d955b6a89be2453fecccf1103/ViomeRecommendations_Demo.pdf.

36
*Id.* at 7–55.

37 Melody J. Slashinski et al., *‘Snake-oil,’ ‘Quack Medicine,’ and ‘Industrially Cultured Organisms’: Biovalue and the Commercialization of Human Microbiome Research*, 13 BMC Med. Ethics, Oct. 2012, at 4.

38
*See* discussion *infra* Section IV.B.

39 Slashinski et al., *supra* note 37, at 6.

40 Gregory L. Damhorst et al., *Current Capabilities of Gut Microbiome-Based Diagnostics and the Promise of Clinical Application*, 223 J. Infectious Disease S270 (2021).

41 Claire M. Doocey et al., *The impact of the human microbiome in tumorigenesis, cancer progression, and biotherapeutic development*, 22 BMC Microbiology, Feb. 12, 2022, at 2.

42
*Id.* at 3.

43
*Id.*

44 J. Raes, *Microbiome-based companion diagnostics: no longer science fiction?*, 65 Gut 896, 896 (2016).

45 Analytical validity is the ability to detect or measure the analyte/component that the test is intended to measure. U.S. Food & Drug Admin., *Direct-to-Consumer Tests* (Dec. 20, 2019), https://www.fda.gov/medical-devices/vitro-diagnostics/direct-consumer-tests [hereinafter *FDA DTC Tests*]. *See also infra* Section III.C.

46 Alan W. Walker & Lesley Hoyles, *Human microbiome myths and misconceptions*, 8 Nat. Microbiol. 1392 (2023). Clinical validity refers to the ability of a test to identify an existing health condition or predict the likelihood that an individual will develop a disease or health condition. *See*  Ctrs. Medicare & Medicaid Servs., CLIA Overview (Oct. 22, 2013), https://www.cms.gov/regulations-and-guidance/legislation/clia/downloads/ldt-and-clia_faqs.pdf [hereinafter CLIA Overview].

47 Alla Katsnelson, *Standards seekers put the human microbiome in their sights*, C&EN (July 14, 2019), https://cen.acs.org/biological-chemistry/microbiome/Standards-seekers-put-human-microbiome/97/i28. *See also* Diane E. Hoffmann et al., *The DTC microbiome testing industry needs more regulation*, 383 Science 1176 (2024); Stephanie L. Servetas et al., *Evaluating the Analytical Performance of Direct-to-Consumer Gut Microbiome Testing Services*, NIST (June 28, 2024), https://www.biorxiv.org/content/10.1101/2024.06.05.596628v2.full.pdf.

48 Companies also market to these two groups. *See eg Diagnostic Sol. Lab.*, *supra* note 25 (stating that ‘almost every patient can benefit from a GI-MAP gut health assessment’ to ‘optimize overall health’ and ‘unravel the root causes of chronic symptoms’).

49 All recruitment materials were approved by the Institutional Review Board of the University of Maryland, Baltimore. For a complete report of the conduct of the patient/consumer focus groups and findings, *see* Jennifer Huang, *Regulation of Microbiome-Based Diagnostic Tests: Alm 2 – Tested and Untested Consumer Focus Group Findings*, Westat (Nov. 11, 2021), https://www.law.umaryland.edu/media/sol/sol-2022-images-and-files/academics/programs-and-centers/health-law-program/pdfs-docs-and-files/Microbiome-Tested-and-Untested-Consumers-Focus-Group-Report.pdf.

50
*Project Overview*, Microsetta Initiative, https://microsetta.ucsd.edu/project-overview/ (last visited July 4, 2025).

51
*See* Jennifer Huang, *Regulation of Microbiome-Based Diagnostic Tests: Aim 2 - Provider Focus Group Findings*, Westat (June 1, 2021) https://www.law.umaryland.edu/media/sol/sol-2022-images-and-files/academics/programs-and-centers/health-law-program/pdfs-docs-and-files/Microbiome-Physician-Focus-Groups-Report.pdf [hereinafter *Physician Focus Group Report*].

52 Functional medicine physicians focus on the root causes of diseases and take a holistic approach to patient care. ‘They provide “patient-centered” care, … learning about [a patient’s] lifestyle, medical history, family history, and needs in order to find a solution to [the patient’s] health problems.’ *What is a Functional Medicine Doctor?*  WebMD (Dec. 18, 2023), https://www.webmd.com/a-to-z-guides/what-is-a-functional-medicine-doctor (stating that not all functional medicine providers are M.D.s; they include D.O.s, naturopaths, chiropractors, and physician assistants).

53 Some of the functional medicine physicians reported that ‘they ordered these tests for 5% of their patients, while others ordered these tests for the majority of their patients. One functional medicine physician stated that she worked with a company that produces microbiome tests and has a fair amount of experience reviewing results. In terms of ordering these tests, functional medicine physicians stated that this was one among a larger panel of tests used to gain a larger understanding of the patient’s gut and overall health.’ *Physician Focus Group Report*, *supra* note 51, at 7.

54
*Id.* at 10.

55
*Id.* at 11.

56
*Id.* at 8.

57
*Id.* at 10.

58
*Id.* at 13.

59
*Id.* at 15.

60
*Id.* at 16.

61
*Id.* at 17.

62
*Id.* at 21.

^63^
Christina Farr, *Insiders describe aggressive growth tactics at uBiome, the health start-up raided by the FBI last week*, CNBC (May 2, 2019), https://www.cnbc.com/2019/05/02/ubiome-what-really-happened-at-health-start-up-raided-by-fbi.html.

64
u Biome, https://ubiome.com/ [https://web.archive.org/web/20190520005828/https:/ubiome.com/].

65
*The World’s Most Innovative Companies 2018 Honorees by Sector*, Fast Co., https://www.fastcompany.com/most-innovative-companies/2018/sectors/data-science (last visited July 4, 2025).

^66^
Erin Brodwin, *I tried a test from troubled poop-testing startup uBiome that let me peek inside a ‘forgotten organ.’ Here’s what I learned.*, Bus. Insider (May 20, 2019), https://www.businessinsider.com/microbiome-gut-bacteria-test-forgotten-organ-future-medicine-ubiome-photos-2018-11.

67 Farr, *supra* note 63.

68
*Id.*

69
*Id.*

70 Alex Keown, *Five Months After FBI Raid, uBiome Files for Bankruptcy*, BioSpace (Sept. 5, 2019), https://www.biospace.com/article/under-fbi-scrutiny-ubiome-files-for-bankruptcy/.

71 Conor Hale, *Bankrupt microbiome startup uBiome to liquidate assets, shutter operations,*  Fierce Biotech (Oct. 2, 2019), https://www.fiercebiotech.com/medtech/bankrupt-microbiome-startup-ubiome-to-liquidate-assets-shutter-operations.

72 Erin Brodwin, *Bankrupt uBiome preliminarily sells patents for 1% of the poop-testing startup’s original valuation*, STAT (Dec. 16, 2019), https://www.statnews.com/2019/12/16/microbiome-startup-ubiome-patents-sold/.

73
*SEC Charges Co-Founders of San Francisco Biotech Company with $60 Million Fraud,* U.S. Sec. & Exch. Comm’n (March 19, 2021), https://www.sec.gov/litigation/litreleases/2021/lr25056.htm.

74
*Id.*

75
*Sec. & Exch. Comm’n v. Richman*, No. 21-CV-01911-CRB, 2021 WL 5113168 (N.D. Cal. Nov. 3, 2021).

76 Hoffmann et al., *supra* note 47, at 1178.

77 Giuseppe Novelli et al., *Genetic tests and genomic biomarkers: regulation, qualification and validation*, 5 Clin. Cases Mineral Bone Metab. 149 (2008).

78
*Id. See also The knowns and unknowns of the human microbiome*, Gut Microbiota for Health by ESNM (Aug. 15, 2019), https://www.gutmicrobiotaforhealth.com/the-knowns-and-unknowns-of-the-human-microbiome/. Approximately 20% of bacterial gene sequences have not been identified, and over 40% of the 10 million microbial genes have uncharacterized functions. *Id.*

79 Hoffmann et al., *supra* note 47, at 1178.

80
*Id.* at 1177.

81 Servetas et al., *supra* note 47.

82
*Id*.

83 Jens Walter et al., *Establishing or Exaggerating Causality for the Gut Microbiome: Lessons from Human Microbiota-Associated Rodents*, 180 Cell 221 (2020). However, evidence of a causal relationship is not necessary for a diagnostic test to be valid. A microorganism could be diagnostic for a disease without contributing to the disease itself. The association may be strong enough to conclude that it is diagnostic—the disease itself could have led to this microorganism starting to grow but it is not responsible for the disease.

84
*See* Tamara Duker Freuman, *5 Reasons to Skip Gut Microbiome Testing – For Now*, FODMAP Everyday (updated June 13, 2025), https://www.fodmapeveryday.com/5-reasons-to-skip-gut-microbiome-testing-for-now/.

85 Hoffmann et al., *supra* note 47, at 1178.

86
*Id.*

87
*See* 42 U.S.C. § 263a; Laboratory Requirements, 42 C.F.R. § 493 (2024).

88
*See infra* Section IV.A.2.

89
*See infra* Section IV.A.5.

90
*See infra* Section IV.A.3.

91
Ctrs. Medicare & Medicaid Servs. *Clinical Laboratory Improvement Amendments (CLIA)* (updated June 24, 2025), https://www.cms.gov/Regulations-and-Guidance/Legislation/CLIA.

92 42 C.F.R. § 493.2 (2024). CLIA does not define diagnosis, prevention, treatment, or health but looks to state law to supply these definitions.

93
*Laboratory Developed Tests,* U.S. Food & Drug Admin. (Feb. 4, 2025), https://www.fda.gov/medical-devices/in-vitro-diagnostics/laboratory-developed-tests [hereinafter *FDA LDTs*]. ‘LDTs are in vitro diagnostic products (IVDs) that are intended for clinical use and are designed, manufactured, and used within a single laboratory that is certified under [CLIA] and meets the regulatory requirements under CLIA to perform high complexity testing.’ *Definitions and General Oversight*, FDA LDTs (Jan. 15, 2025), https://www.fda.gov/medical-devices/laboratory-developed-tests-faqs/definitions-and-general-oversight-laboratory-developed-tests-faqs.

94 CLIA Overview, *supra* note 46.

95
*Consumer Direct Access to Laboratory Testing*, ASCLS (July 1, 2021), https://ascls.org/direct-access-testing-2021/.

96 Such analytical validation is to include an ‘analysis of accuracy, precision, analytical sensitivity, analytical specificity, reportable range, reference interval, and any other performance characteristics required for the test system in the laboratory that intends to use it.’ CLIA Overview, *supra* note 46 at 3. *See also* 42 C.F.R. § 493.1253 (2024).

97 CLIA Overview, *supra* note 46, at 1 (‘CMS’s CLIA program does not address the clinical validity of any test.’).

98
Ctrs. Disease Control & Prevention, *Clinical Laboratory Improvement Amendments (CLIA) Test Complexities* (Sept. 11, 2024), https://www.cdc.gov/clia/php/test-complexities/index.html [hereinafter *CLIA Test Complexities*]. FDA is responsible for determining the complexity level of a given type of testing. *See*  Nat’l Hum. Genome Rsch. Inst., The CLIA Framework (last visited Sept. 29, 2024), https://www.genome.gov/Pages/PolicyEthics/GeneticTesting/The_CLIA_Framework.pdf [hereinafter CLIA Framework]. More complicated tests require stricter CLIA quality standards requirements, personnel qualifications, and responsibilities. *See*  Ctrs. Disease Control & Prevention, CLIA Program & Medicare Lab Services (Dec. 2024), https://www.cms.gov/Outreach-and-Education/Medicare-Learning-Network-MLN/MLNProducts/Downloads/CLIABrochure.pdf [hereinafter CLIA Program & Medicare Lab Services].

99 Proficiency Testing (PT) ‘is the testing of unknown samples sent to a laboratory by an HHS-approved PT program…. After testing, the laboratory reports its sample results back to their PT program. The program grades the results using the CLIA grading criteria and sends the laboratory their scores.’ Ctrs. Medicare & Medicaid Servs., Clinical Laboratory Improvement Amendments (CLIA): Proficiency Testing and PT Referral at 2 (Oct. 2024) https://www.cms.gov/Regulations-and-Guidance/Legislation/CLIA/Downloads/CLIAbrochure8.pdf. While many laboratories are required to perform PT, there are no proficiency testing programs for some categories of tests, eg genetic tests. Personalized Med. Coal., Policy Brief: CLIA and Genetic Testing (last visited July 4, 2025), https://www.personalizedmedicinecoalition.org/Userfiles/PMC-Corporate/file/pmc_policy_brief_CLIA_genetic_testing.pdf.

100 CLIA Program & Medicare Lab Services, *supra* note 98.

101
*CLIA Test Complexities*, *supra* note 98.

102 CLIA Overview, *supra* note 46.

103
*See* 42 C.F.R. § 493.2 (2024); *see also* CLIA Framework, *supra* note 98.

104
*See* U.S. Food & Drug Admin., Optimizing FDA’s Regulatory Oversight of Next Generation Sequencing Diagnostic Tests – Preliminary Discussion Paper (Dec. 29, 2014), https://www.fda.gov/media/90403/download; *see also FDA DTC Tests*, *supra* note 45; CLIA Framework, *supra* note 98.

105
*See* CLIA Framework, *supra* note 98.

106
*See* 42 C.F.R. § 493 (2024).

107
*See id.*

108 Novelli et al., *supra* note 77.

109 21 U.S.C. § 321 (2024). The term ‘device’ does not include software functions as described in 21 U.S.C. § 360j (2024). Furthermore, the quoted definition excludes parts of the definition we deem not relevant to the categorization of DTC microbiome tests.

110 For a product to be Class I, it must *not* be (1) invasive, (2) implanted, or (3) ‘pose a risk to the safety of users and other persons if specific regulatory controls are not applied.’ U.S. Food & Drug Admin., General Wellness: Policy for Low Risk Devices – Guidance for Industry and Food and Drug Administration Staff 5 n.9 (2019), https://www.fda.gov/media/90652/download [hereinafter 2019 Low Risk Devices Industry Guidance].

111 General controls further require that a device manufacturing facility register with FDA and provide FDA with a list of all devices it plans to market. *See Regulatory Controls*, U.S. Food & Drug Admin. (Mar. 27, 2018), https://www.fda.gov/medical-devices/overview-device-regulation/regulatory-controls.

112 Types of special controls include performance standards, post-market surveillance, and special labeling requirements. *Id.*

113
*Id.*

114 Elizabeth Mansfield et al., *Food and Drug Administration Regulation of In Vitro Diagnostic Devices*, 7 J. Molecular Diagnostics 2, 3 (2005). Examples of Class II IVD tests are molecular tests for prothrombin G20210A and factor V Leiden, which are conditions that increase one’s chances of serious blood clots; an example of a Class III IVD test is a Pap smear digital image analysis system. *Id.*

115 A medical device is considered an IVD when (1) it is intended for use in the diagnosis of a disease or condition; (2) test specimens are taken from the human body; and (3) the test can be used in a laboratory, doctor’s office, or at home. Kimberly Piermatteo, *Is My Product a Medical Device?*, U.S. Food & Drug Admin., https://www.fda.gov/media/131268/download (last visited July 4, 2025).

116
*Overview of IVD Regulations,* U.S. Food & Drug Admin. (Dec. 20, 2024), https://www.fda.gov/medical-devices/ivd-regulatory-assistance/overview-ivd-regulation.

117
*In-Vitro Diagnostics,* U.S. Food & Drug Admin., (Nov. 13, 2024), https://www.fda.gov/medical-devices/products-and-medical-procedures/vitro-diagnostics; 21 C.F.R. § 809, *In Vitro Diagnostic Products for Human Use*.

118 U.S. Food & Drug Admin., DEN160026, Letter to 23andME, Inc. on 23andMe Personal Genome Service (PGS) Test Evaluation of Automatic Class III Designation – De Novo Request (Nov. 2, 2017), https://www.accessdata.fda.gov/cdrh_docs/pdf16/den160026.pdf [hereinafter FDA Letter to 23andMe].

119
*Differences Between the 510(k) and PMA Approval Processes and How this Affects Lawsuits* (Apr. 29, 2019), https://cowperlaw.com/differences-between-the-510k-and-pma-approval-processes-and-how-this-affects-lawsuits/.

120
*De Novo Classification Request,* U.S. Food & Drug Admin. (Oct. 4, 2022), https://www.fda.gov/medical-devices/premarket-submissions/de-novo-classification-request [hereinafter *FDA De Novo Classification*]. The *de novo* pathway is appropriate when general controls alone or general and special controls provide sufficient assurance that the device is safe and effective for its intended use. *Id.*

121
*Id.*

122
*Learn if a Medical Device Has Been Cleared by FDA for Marketing,* U.S. Food & Drug Admin. (Dec. 29, 2017), https://www.fda.gov/medical-devices/consumers-medical-devices/learn-if-medical-device-has-been-cleared-fda-marketing.

123
*Premarket Approval (PMA)*, U.S. Food & Drug Admin. (May 16, 2019), https://www.fda.gov/medical-devices/premarket-submissions-selecting-and-preparing-correct-submission/premarket-approval-pma.

124
*FDA DTC Tests*, *supra* note 45.

125 FDA’s position regarding LDTs evolved largely in response to DTC genetic tests. Traditionally, LDTs were simple, in-house tests designed to measure a single analyte (eg hemoglobin A1C), and FDA excluded them from medical device regulation as the Agency did not consider clinical laboratories medical device manufacturers. In the 1990s, the FDA changed that policy and adopted a policy of enforcement discretion—that it had the authority to regulate LDTs because clinical laboratories can be manufacturers, but it had discretion not to enforce its regulations and, as a policy matter, largely would not do so. However, the FDA has regulated LDTs when the Agency felt there was a high risk. Bruce R. Parker & Casey L. Bryant, *Potential FDA Regulation of Laboratory-Developed Tests and the Effect on Litigation*, For the Def. 65, 67 (2018). *See also FDA LDTs*, *supra* note 93.

126 On Apr. 29, 2024, FDA announced a final rule amending its regulations ‘to make explicit that in vitro diagnostic products (IVDs) are devices under the Federal Food, Drug, and Cosmetic Act (FD&C Act) including when the manufacturer of the IVD is a laboratory.’ *FDA LDTs*, *supra* note 93. The Federal Register statement announcing the new rule made clear that, FDA would ‘provide greater oversight of … LDTs through a phaseout of its general enforcement discretion approach … over the course of four years, as well as targeted enforcement discretion policies for certain categories of IVDs manufactured by laboratories.’ Medical Devices; Laboratory Developed Tests, 89 Fed. Reg. 37286 (May 6, 2024) (to be codified at 21 C.F.R. pt. 809).

127 Am. Clin. Lab. Ass’n, 2025 WL 964238, at ^*^13. *See also Federal Court Vacates FDA Rule on Laboratory Developed Testing Services, Siding with ACLA*, ACLA (Mar. 31, 2025), https://www.acla.com/federal-court-vacates-fda-rule-on-laboratory-developed-testing-services-siding-with-acla/. The court also relied on the ‘major questions doctrine’ in arriving at its decision. *Id.*

128 This belief is based on the fact that the ‘former Trump administration took the position in a June 2020 memorandum that the FDCA does not give the FDA the authority to regulate LDTs.’ Alissa D. Fleming, Robert E. Mazer & Mary Grace Griffin, *Federal Court Vacates LDT Final Rule*, Baker Donelson (Apr. 11, 2025), https://www.bakerdonelson.com/federal-court-vacates-ldt-final-rule.

129 Am. Clin. Lab. Ass’n, 2025 WL 964238, at ^*^1; *see also* ‘A “laboratory-developed test” is a methodology or process by which a laboratory generates biochemical, genetic, molecular, or other forms of clinical information about a patient specimen *for use by the treating physician*.’ *Id.* at ^*^3 (emphasis added).

130
*See* final FDA regulation, 89 Fed. Reg. 37286, 37331 (May 6, 2024) (rescinded by 90 Fed. Reg. 63912 (Sept. 19, 2025)), stating ‘The Cures Act, enacted in 2016, amended the FD&C Act to exclude certain software functions from the statutory “device” definition unless certain criteria are met. See 21 U.S.C. 360j(o). Congress would have had no need to make this amendment to the FD&C Act if the device definition did not already cover software, which is a thing that cannot be “touched and held.” This underscores that a plain reading of the device definition may include things that cannot be “touched and held.”’ *See also* Deborah Levenson, *District court strikes down the FDA’s final laboratory developed tests rule,* ADLM (Apr. 24, 2025), https://myadlm.org/cln/articles/2025/mayjune/district-court-strikes-down-the-fdas-final-laboratory-developed-tests-rule#:~:text=Reactions%20from%20labs%20and%20legal,their%20own%20assays%2C%20he%20explained.

131 Kashal T. Kadalakia et al., *Public Health Implications of Legal Challenges to the FDA’s Regulation of Laboratory-Developed Tests*, JAMA Health Forum (June 20, 2025).

132 The Agency defines these tests as IVDs ‘that are marketed directly to consumers without the involvement of a health care provider.’ *FDA DTC Tests*, *supra* note 45.

133 89 Fed. Reg. at 37407.

134
*See FDA DTC Tests*, *supra* note 45.

135
*Id.*

136
*Id. See also* 2019 Low Risk Devices Industry Guidance, *supra* note 110, at 2.

137
*See* General Wellness: Policy for Low Risk Devices; Draft Guidance for Industry and Food and Drug Administration Staff, 80 Fed. Reg. 2712 (Jan. 1, 2015) and 81 Fed. Reg. 49993 (July 29, 2016).

138 2019 Low Risk Devices Industry Guidance, *supra* note 110.

139 The 2016 Low Risk Devices Industry Guidance was subsequently updated in 2019, after the passage of the 21st Century Cures Act, Pub. L. No. 114-255 (2016). *See also infra* notes 152–56 and accompanying text.

140 2019 Low Risk Devices Industry Guidance, *supra* note 110, at 5.

141 Examples of products that would be considered low risk include a software function that reminds users to keep exposed skin out of direct sunlight; a portable product that is intended to monitor the pulse rate of users during exercise; a software function that monitors and records food consumption. *Id.* at 7–8.

142
*FDA DTC Tests*, *supra* note 45.

143
*Id.*

144
*23andMe Gets FDA Clearance to Market Bloom Syndrome Carrier Test Directly to Consumers*, GenomeWeb (Feb. 19, 2015), https://www.genomeweb.com/molecular-diagnostics/23andme-gets-fda-clearance-market-bloom-syndrome-carrier-test-directly.

145 These tests, unlike traditional diagnostic tests that tell a patient whether they currently have a disease, tell consumers about their chance of developing a disease in the future. *FDA DTC Tests*, *supra* note 45.

146
*FDA Allows Marketing of First Direct-to-Consumer Tests that Provide Genetic Risk Information for Certain Conditions*, U.S. Food & Drug Admin. (Apr. 6, 2017),
https://web.archive.org/web/20190518001549/https://www.fda.gov/news-events/press-announcements/fda-allows-marketing-first-direct-consumer-tests-provide-genetic-risk-information-certain-conditions.

147 Allyse et al., *supra* note 3 at 113.

148 FDA Letter to 23andMe, *supra* note 118.

149
*Id.*

150
*FDA Authorizes First DTC Cancer Risk Test, 23andMe’s GRH Report for BRCA1 and BRCA2 Mutations*, Inside Precis. Med. (Mar. 6, 2018), https://www.insideprecisionmedicine.com/news-and-features/fda-authorizes-first-dtc-cancer-risk-test-23andmes-grh-report-for-brca1-and-brca2-mutations/#:~:text=The%20FDA%20has%20granted%2023andMe%20the%20agency%E2%80%99s%20first,most%20common%20in%20people%20of%20Ashkenazi%20Jewish%20descent.

151
*FDA DTC Tests*, *supra* note 45.

152 FDA’s policy regarding general wellness devices is based on its interpretation of Section 3060(a) of the 21st Century Cures Act which removed general wellness support software from the definition of medical device. 21 U.S.C. § 360j.

153 The purpose of the legislation was to expedite the marketing of new medical device therapies and diagnostics.

154 21 U.S.C. § 360.

155 2019 Low Risk Devices Industry Guidance, *supra* note 110.

156
*Id.* at 1.

157
*Id.* at 3.

158 A disease or condition ‘means damage to an organ, part, structure, or system of the body such that it does not function properly (e.g., cardiovascular disease), or a state of health leading to such dysfunction (e.g., hypertension); except that diseases resulting from essential nutrient deficiencies (e.g., scurvy, pellagra) are not included in this definition.’ 21 C.F.R. § 101.14(a)(5) (2024).

159 2019 Low Risk Devices Industry Guidance, *supra* note 110, at 3.

160
*Id.* at 4.

161
*Id.*

162
*Id.* at 4–5.

163
*Id.* at 5. The Guidance further elaborates by stating that ‘such associations are described in peer-reviewed scientific publications or official statements made by healthcare professional organizations.’ *Id.*

164
*Id.* at 4.

165 The recent *Am. Clin. Lab. Ass’n v. FDA* decision leaves the significance of these exemptions under the 21st Century Cures Act unclear. Virtually all, if not all, of the DTC microbiome tests use software (as do many LDTs) to analyze assays and make recommendations. If software is considered not tangible, under the Am. Clin. Lab. Ass’n ACLA decision, under the reasoning of the Court, all software-based tests could be considered exempt from medical device regulation. This would have a much broader impact than simply exempting LDTs from regulation as medical devices and seems to directly contradict Congress’s understanding of what has been subject to medical device regulation under the 21st Century Cures Act. *See* Rachel E. Sachs, Joshua M. Sharfstein, and Patricia J. Zettler, *Judicial Invalidation of the FDA’s Laboratory-Developed Test Rule—Legal and Public Health Consequences*, NEJM (May 7, 2025).

166 Section 520 (o) (1)(A)-(D) of the FD&C Act (‘The 21st Century Cures Act’) states, The term device,… shall not include a software function that is intended—(A) for administrative support of a health care facility, including the processing and maintenance of financial records, claims or billing information, appointment schedules, business analytics, information about patient populations, admissions,…; (B) for maintaining or encouraging a healthy lifestyle and is unrelated to the diagnosis, cure, mitigation, prevention, or treatment of a disease or condition; (C) to serve as electronic patient records, including patient-provided information, to the extent that such records are intended to transfer, store, convert format, or display the equivalent of a paper medical chart, so long as—(i) such records were created, stored, transferred, or reviewed by health care professionals, or by individuals working under supervision of such professionals; (ii) such records are part of health information technology…; and (iii) such function is not intended to interpret or analyze patient records, including medical image data, for the purpose of the diagnosis, cure, mitigation, prevention, or treatment of a disease or condition; (D) for transferring, storing, converting formats, or displaying clinical laboratory test or other device data and results, findings by a health care professional with respect to such data and results, general information about such findings, and general background information about such laboratory test or other device, unless such function is intended to interpret or analyze clinical laboratory test or other device data, results, and findings. FD&C Act § 520(o)(1(E)) further exempts from medical device regulation, software functions intended to provide decision support for the diagnosis, treatment, prevention, cure, or mitigation of disease or other conditions (often referred to as clinical decision support CDS software). *Id.*

167
*Id.*

168
*See generally*  Multiple Function Device Products: Policy and Considerations – Guidance for Industry and Food and Drug Administration Staff, U.S. Food & Drug Admin. (July 29, 2020), https://www.fda.gov/media/112671/download [hereinafter Multiple Function Device Industry Guidance] (stating that FDA considers a multiple function device as one that has both device and non-device functions).

169 ‘Other functions’ are non-device functions and, as such, are not subject to the premarket review of safety and effectiveness that device functions must go through. *Id.* at 5.

170 § 3060(a), 21 U.S.C. § 301 *et seq*.

171
Multiple Function Device Industry Guidance, *supra* note 168, at 5. ‘For example, if a manufacturer seeks clearance or approval for a device function (e.g., analysis), and not the device function for which FDA has expressed its intention not to enforce compliance (e.g., trend), then FDA intends to only review the analysis function and assess the trend function only insofar as it could negatively impact the analysis function. In that instance, because FDA is reviewing the analysis function only, FDA’s decision to clear or approve applies only to the analysis function.’ *Id*. at 6.

172
*Id.* at 8.

173 FDA’s Multiple Function Device Industry Guidance states explicitly that in determining if an ‘other function’ will impact the safety and/or effectiveness of the device function, a manufacturer should ask: ‘Does the “other function” provide input data that is used for a critical calculation within the device function-under-review? Does the device function-under-review rely on results from the “other function?”’ *Id.* at 10.

174 Section 3060(a), 21 U.S.C. § 301 *et seq*.

175
*Id.* However, if a company did not provide recommendations and its test only conducted functions 1–4, there might be a valid argument that it does meet the exemption, but that would depend on whether determining the composition of the sample was considered a device function because it ‘interprets or analyzes’ device data.

176 FD&C Act § 520(o).

177
*Id.*

178 Barbara J. Evans et al., *How Can Law and Policy Advance Quality in Genomic Analysis and Interpretation for Clinical Care?*, 48 J.L. Med. & Ethics 44, 57 (2020).

179
*Physician Focus Group Report*, *supra* note 51.

180
*See eg Diagnostic Sol. Lab*, *supra* note 25, *and* Doctor’s Data, *GI360*; *stool*  https://www.doctorsdata.com/GI360-stool (last visited July 4, 2025).

181
Clinical Decision Support Software: Guidance for Industry and Food and Drug Administration Staff, U.S. Food & Drug Admin. at 4, n.1 (Sept. 28, 2022), https://www.fda.gov/media/109618/download [hereinafter CDS Industry Guidance].

182
*Nutritionist, Licensed Nutritionist, and Registered Dietitian Requirements by State*, nutritionEd.org, https://www.nutritioned.org/state-requirements/ (last visited July 4, 2025).

183
Md. Code, Health Occ. § 5-101(h)(2)(v) (2024).

184 CDS Industry Guidance, *supra* note 181, at 6.

185
*Id.*

186 For example, software such as the software used in the 23andMe GHR test queries a genomic reference database to make CDS recommendations. FDA asserted in 2019 draft guidance that, because such CDS software recommendations are not transparent to users (ie are based on black box software), the Agency can regulate most SaMD under the 21st Century Cures Act. *Id.* at 6–7.

187
*Id.* at 7.

188 Dan Kagan et al. *Your clinical decision support software may now be regulated by FDA as a medical device*, DLA Piper (Sept. 29, 2022), https://www.dlapiper.com/en/insights/publications/2022/09/your-clinical-decision-support-software-may-now-be-regulated-by-fda-as-a-medical-device. *See supra* note 109 and accompanying text.

189 CDS Industry Guidance, *supra* note 181, at 8. ‘For Next Generation Sequencing (NGS), a fluorescent signal on tagged DNA is processed by modification or transformation into base pairs and sequences. Genetic sequences, including datasets of sequence variants that differ from reference sequences and datasets filtered to represent disease-associated variations (such as variant call format files or VCFs), are examples of patterns.’ *Id.*

190
*Id.*

191 This would be the case if FDA interprets the *Am. Clin. Lab. Ass’n v. FDA* opinion in a way that would allow it to continue to regulate DTC tests as medical devices or Congress passes legislation overturning the decision or clarifying a carveout for DTC tests.

192 This statement assumes that, going forward, FDA is able to regulate DTC software-based LDTs as medical devices under revised regulations or under a new statutory provision. *See* Levenson, *supra* note 130.

193 In fact, it is the manufacturer who makes this decision as there is no requirement that manufacturers submit an application for premarket review or consult with FDA prior to marketing their test. It is only where FDA has some indication that the product is being illegally marketed that it would make this assessment.

194
*See supra* notes 152–63 and accompanying text.

195 U.S. Food & Drug Admin., *General Controls for Medical Devices* (Dec. 15, 2023), https://www.fda.gov/medical-devices/regulatory-controls/general-controls-medical-devices#misbranding [hereinafter *General Controls for Medical Devices*].

196 Bill Sutton, *Overview of Regulatory Requirements: Medical Devices – Transcript*, U.S. Food & Drug Admin. (Jan. 4, 2018), https://www.fda.gov/training-and-continuing-education/cdrh-learn/overview-regulatory-requirements-medical-devices-transcript.

197
*General Controls for Medical Devices*, *supra* note 195.

198
*Introduction to Medical Device Labeling,* U.S. Food & Drug Admin. (Oct. 23, 2020), https://www.fda.gov/medical-devices/overview-device-regulation/device-labeling [hereinafter *Device Labeling*].

199 21 U.S.C. § 352 (2024). A restricted device is one for which FDA requires the authorization of a licensed practitioner to sell, distribute, or use because there cannot be reasonable assurance of its safety and efficacy. *See General Controls for Medical Devices*, *supra* note 195; *see also* 21 U.S.C. § 360j (2024).

200 Scott S. Liebman & Jessica B. Lee, *Safe and Effective! Developing FDA-compliant Advertising and Promotions for Drugs and Medical Devices* at 7 (2017), https://www.loeb.com/~/media/files/pdfs/loeb%20social%20media_life%20sciences%20presentation.pdf.

201 U.S. Food & Drug Admin., *Providing Regulatory Submissions in Electronic and Non-Electronic Format—Promotional Labeling and Advertising Materials for Human Prescription Drugs, Guidance for Industry* 2 (2022), https://www.fda.gov/media/128163/download.

202 Liebman & Lee, *supra* note 200, at 20.

203 U.S. Food & Drug Admin., *Guidance for Industry: Presenting Risk Information in Prescription Drug and Medical Device Promotion* 3–4 (2009), https://www.fda.gov/media/76269/download [hereinafter *Risk Information Industry Guidance*].

204 Warning Letter to Ramaswamy Iyer, Director, Inova Genomics Lab., MARCS-CMS 577422 (Food & Drug Admin. Apr. 4, 2019), https://www.fda.gov/inspections-compliance-enforcement-and-criminal-investigations/warning-letters/inova-genomics-laboratory-577422-04042019.

205
*Id.*

206
*Id.*

207
*Id.*

208 Federal Trade Commission Act 15 U.S.C. §§ 41–58 (2024).

209
*Truth in Advertising*, Fed. Trade Comm’n (last visited Oct. 6, 2024), https://www.ftc.gov/news-events/media-resources/truth-advertising.

210 Liebman & Lee, *supra* note 200, at 22.

211
*Id.* at 24.

212
*FTC Policy Statement Regarding Advertising Substantiation*, Fed. Trade Comm’n (Nov. 23, 1984), https://www.ftc.gov/public-statements/1984/11/ftc-policy-statement-regarding-advertising-substantiation. *See also Health Products Compliance Guidance,*  Fed. Trade Comm’n (Dec. 2022), https://www.ftc.gov/system/files/ftc_gov/pdf/Health-Guidance-508.pdf

213
*Companies Pitching Genetically Customized Nutritional Supplements will Drop Misleading Disease Claims*, Fed. Trade Comm’n (Jan. 7, 2014), https://www.ftc.gov/news-events/press-releases/2014/01/companies-pitching-genetically-customized-nutritional-supplements [hereinafter *FTC GeneLink Press Release*].

214
*Id.*

215
*See DTC Microbiome-Based Testing Companies*, *supra* note 24.

216
*Id*.

217
*Id.*

218 A misleading claim is considered misbranding under 21 C.F.R. pt. 801 (2024). *See also Device Labeling*, *supra* note 198 and accompanying text.

219 21 C.F.R. § 101.93 (2024).

220 Under DSHEA, companies are responsible for the safety and proper labeling of dietary supplements, like probiotics, before they enter the marketplace. *Dietary Supplements*, U.S. Food & Drug Admin. (Oct. 1, 2024), https://www.fda.gov/food/dietary-supplements.

221 Dietary supplements may make structure/function claims without premarket approval and health claims (reduction of risk of disease claims), if supported by scientific evidence. *See Structure/Function Claims*, U.S. Food & Drug Admin. (Mar. 28, 2024), https://www.fda.gov/food/food-labeling-nutrition/structurefunction-claims; *see also Guidance for Industry: Evidence-Based Review System for the Scientific Evaluation of Health Claims*, U.S. Food & Drug Admin. (Sept. 17, 2018), https://www.fda.gov/regulatory-information/search-fda-guidance-documents/guidance-industry-evidence-based-review-system-scientific-evaluation-health-claims. Health claims must be reviewed and evaluated by FDA prior to marketing the product. *Guidance for Industry: Notification of a Health Claim or Nutrient Content Claim Based on an Authoritative Statement of a Scientific Body*, U.S. Food & Drug Admin. (Sept. 20, 2018), https://www.fda.gov/regulatory-information/search-fda-guidance-documents/guidance-industry-notification-health-claim-or-nutrient-content-claim-based-authoritative-statement. 2019 Low Risk Devices Industry Guidance  *supra* note 110, at 4.

222 2019 Low Risk Devices Industry Guidance *supra* note 110, at 4.

223 FDA uses the reasonable consumer standard in determining whether a claim is misleading. *Risk Information Industry Guidance*, *supra* note 203, at 5.

224 Joel C. Eissenberg, *Direct-to-Consumer Genomics: Harmful or Empowering?*, 114 Mo. Med. 26, 29 (2017).

225 In March 2025, 23andMe filed for bankruptcy. The filing was not a surprise given its financial difficulties, a major data breach exposing the personal information of several million customers, and a related lawsuit. Max Eddy, *23andMe just filed for bankruptcy. You should delete your data now.*, NYT/Wirecutter (March 25, 2025), https://www.nytimes.com/wirecutter/reviews/23andme-data-bankrupt/.

226
*See eg* Scott Bigman, *Revolutionizing Gut Health: The Rise of Microbiome Testing Companies and Their Impact on Personalized Medicine*, Rupa Health (updated Feb. 24, 2025), https://www.rupahealth.com/post/revolutionizing-gut-health-the-rise-of-microbiome-testing-companies-and-their-impact-on-personalized-medicine (stating that ‘Viome [a market leader in DTC microbiome testing] hopes that the data they receive will fuel more research and generate more insight. Like genealogy company 23andMe, Viome is looking to capitalize on the data, possibly selling it to drug manufacturers.’).

227 2016 O.J. (L 119/1) General Data Protection Regulation.

228 Kimberly A. Houser & W. Gregory Voss, *GDPR: The End of Google and Facebook or a New Paradigm in Data Privacy?*, 25 Rich. J.L. & Tech. 1, 11 (2018).

229
*Data Protection Laws of the World*, DLA Piper (Jan. 29, 2023), https://www.dlapiperdataprotection.com/?t=law&c=US; Neil Richards et al., *Understanding American Privacy*, *in*  Research Handbook on Privacy and Data Protection Law: Values, Norms, and Global Politics 2 (Gloria González, Rosamunde Van Brakel, & Paul De Hert, eds., 2022).

230 Examples of privacy laws that apply only to the government are the Privacy Act of 1974, the Freedom of Information Act, and the Foreign Intelligence Surveillance Act. Examples of privacy laws that apply to both the government and private entities are the Electronic Communications Privacy Act and the Computer Fraud and Abuse Act. Richards, *supra* note 229, at 6–10.

231
*Id.* at 10.

232 Health Insurance Portability and Accountability Act of 1996, Pub. L. No. 104-191 (1996).

233
*HIPAA Basics For Providers: Privacy, Security, and Breach Notification Rules*, Ctrs. Medicare & Medicaid Servs. at 3 (Feb. 2023), https://www.cms.gov/Outreach-and-Education/Medicare-Learning-Network-MLN/MLNProducts/Downloads/HIPAAPrivacyandSecurity.pdf.

234 Katherine Drabiak, *Read the Fine Print Before Sending Your Spit to 23andMe*, Hastings Ctr. (Feb. 26, 2016), https://www.thehastingscenter.org/response-to-call-for-essays-read-the-fine-print-before-sending-your-spit-to-23andme-r/.

235 Emily B. Sklar, *Be Careful Where You Spit: Do HIPAA-Covered Genetic Tests Actually Provide Greater Privacy Protection to Consumers?*, 44 Seton Hall Legis. J. 177, 184 (2020).

236 Florian Schaub et al., *A Design Space for Effective Privacy Notices*, *in*  The Cambridge Handbook of Consumer Priv. 366 (Evan Selinger et al. eds., 2018).

237 Rebecca Lipman, *Online Privacy and the Invisible Market for Our Data*, 120 Pa. State L. Rev. 777, 796–97 (2016).

238 Jessica L. Roberts & Jim Hawkins, *When health tech companies change their terms of service*, 367 Science 745, 745 (2020).

239
*Id.* at 746.

240 Solon Barocas & Helen Nissenbaum, *On Notice: The Trouble with Notice and Consent* 2, 5 (2009), https://nissenbaum.tech.cornell.edu/papers/ED_SII_On_Notice.pdf; *see also* Omer Tene & Jules Polonetsky, *Privacy in the Age of Big Data: A Time for Big Decisions*, 64 Stan. L. Rev. Online 63, 68 (2012).

241 Allyse et al., *supra* note 3, at 116.

242
*Table of State Statutes Related to Genomics*, Nat’l Hum. Genome Rsch. Inst. (Jan. 23, 2020), https://www.genome.gov/sites/default/files/media/files/2020-01/table_state_statutes_genomics_2.pdf.

243
*FTC GeneLink Press Release*, *supra* note 213.

244
*Id.*

245 45 C.F.R. pt. 46 (2018); *see also Federal Policy for the Protection of Human Subjects (‘Common Rule’)*, U.S. Dep’t Health & Hum. Servs. (updated June 5, 2025), https://www.hhs.gov/ohrp/regulations-and-policy/regulations/common-rule/index.html.

246 Valerie Gutmann Koch & Kelly Todd, *Research Revolution or Status Quo?: The New Common Rule and Research Arising from Direct-to-Consumer Genetic Testing*, 56 Hous. L. Rev. 81, 105 (2018) (‘If a company intends to bring a product to market,… the research may be subject to the Food and Drug Administration’s (FDA) human subject protection requirements, which are similar to those enumerated in the Common Rule.’) (citing *Comparison of FDA and HHS Human Subject Protection Regulations*, U.S. Food & Drug Admin. (Mar. 13, 2018), https://www.fda.gov/ScienceResearch/SpecialTopics/RunningClinicalTrials/EducationalMaterials/ucm112910.htm).

247 Gutmann Koch & Todd, *supra* note 246, at 96.

248 Liza Dawson, *The Common Rule and Research with Data, Big and Small*, *in*  Big Data, Health Law, and Bioethics 239 (I. Glenn Cohen et al. eds., 2018).

249 Gutmann Koch & Todd, *supra* note 246, at 97.

250 Genetic Information Nondiscrimination Act of 2008, 42 U.S.C. § 2000ff.

251
*Genetic Information Nondiscrimination Act of 2008*, Geo. L. Libr. (Feb. 28, 2022), https://web.archive.org/web/20220322055347/https://guides.ll.georgetown.edu/c.php?g=592919&p=4230130.

252
*Id.*

253
*Id.* GINA protections do not apply to other types of insurers (eg life or disability insurers).

254
*Genetic Discrimination*, Nat’l Hum. Genome Res. Inst. (Jan. 6, 2022), https://www.genome.gov/about-genomics/policy-issues/Genetic-Discrimination.

255
*Table of State Statutes Related to Genomics*, *supra* note 242.

256
*Id.*

257 45 C.F.R. § 146.122; (2009).

258
*See* Alexander Khoruts, *Is fecal microbiota transplantation a temporary patch for treatment of Clostridium difficile infection or a new frontier of therapeutics?*, 12 Expert Rev. Gastroenterology & Hepatology 435, 437 (2018) (asserting that human gut microbiota should be considered ‘human’ as it has co-evolved with the human host and is taxonomically distinct even from that of other primates). *But see* Rachel E. Sachs & Carolyn A. Edelstein, *Ensuring the safe and effective FDA regulation of fecal microbiota transplantation*, 2 J.L. Biosci. 396, 411 (2015) (noting that, according to the Tissue Reference Group within FDA, because transplantation of fecal microbiota ‘does not require… “*human* cells or tissue”, but that it rather requires the transplantation of *microbial* cells or tissue,’ it does not meet the definition of ‘human tissue’).

259 A vaginal or buccal swab is mostly made up of human cells; they can represent >99% of the DNA sequenced by metagenomics. One vaginal testing company, for example, uses metagenomics for their analysis of vaginal samples and, technically, the company would be able to genotype their consumers, obtain their ancestry, and, if the consumer performs repeat sampling analysis, generate a complete genome of the consumer, which would be more information than generated by the 23andMe tests.

260 Sahar Takshi, *Unexpected Inequality: Disparate-Impact from Artificial Intelligence in Healthcare Decisions*, 34 J.L. Health 215, 228 (2021).

261 Natalie Ram et al., *Genealogy databases and the future of criminal investigation*, 360 Science 1078, 1078 (2018); Sarah Zhang, *How a Genealogy Website Led to the Alleged Golden State Killer*, The Atlantic (Apr. 27, 2018), https://www.theatlantic.com/science/archive/2018/04/golden-state-killer-east-area-rapist-dna-genealogy/559070/.

262 Yonghui Ma et al., *Help, Hope and Hype: Ethical Considerations of Human Microbiome Research and Applications*, 9 Protein & Cell 404, 407 (2018).

263 The microbial cloud refers to the millions of bacteria that come from and surround each person. James Gallagher, *Everyone has a ‘microbial cloud’*, BBC News (Sept. 23, 2015), https://www.bbc.com/news/health-34314065.

264 Daria Shamarina et al., *Communicating the Promise, Risks, and Ethics of Large-Scale, Open Space Microbiome and Metagenome Research*, 5 Microbiome 1, 4 (2017).

265 Nicole Farmer et al., *Neighborhood Environment Associates with Trimethylamine-N-Oxide (TMAO) as a Cardiovascular Risk Marker*, 18 Int’l J. Env’t Rsch. & Pub. Health 4296, 4296 (2021).

266
*See eg* Ayesha K. Rasheed, *Personal Genetic Testing and the Fourth Amendment*, 2020 U. Ill. L. Rev. 1249, 1254–55 (2020).

267 U.S. Const. amend. IV.

268 GAO Report*, supra* note 11, at 5–8.

269 Servetas et al., *supra* note 47.

270 GAO Report*, supra* note 11, at 8 (quoting a genetic expert).

271 Robert Schlaberg, *Microbiome Diagnostics*, 66 Clin. Chem.68, 72–73 (2020).

272
*See supra* note 127 and accompanying text.

273 Aparna Nathan, *Setting Standards for Stool*, The Scientist (Jan. 19, 2024), https://www.the-scientist.com/setting-standards-for-stool-71597.

274
*Id.*

275 Hoffmann et al., *supra* note 47.

276
*See* FDA Letter to 23andMe, *supra* note 118 for these requirements and others.

277 FTC also targeted two large food/probiotic companies for enforcement action. In 2010–2011, it charged the Dannon Company and Nestlé HealthCare Nutrition with making unsubstantiated claims about their probiotics products. *See Nestlé HealthCare Nutrition, Inc.*, Fed. Trade Comm’n (Jan. 18, 2011), https://www.ftc.gov/enforcement/cases-proceedings/092-3087/nestle-healthcare-nutrition-inc; *Dannon Company, Inc.*, Fed. Trade Comm’n (Feb. 4, 2011), https://www.ftc.gov/enforcement/cases-proceedings/0823158/dannon-company-inc.

278 Elisabeth Nations, *Direct-To Consumer Genetic Testing Companies: Is Genetic Data Adequately Protected in the Absence of HIPPA?*, Univ. S. Cal. Gould Bus. L. Digest (Jan. 19, 2023), https://lawforbusiness.usc.edu/direct-to-consumer-generic-testing-companies-is-genetic-data-adequately-protected-in-the-absence-of-hippa/; *See also* Catherine Roberts, *The Privacy Problems of Direct-to-Consumer Genetic Testing*, Consumer Rep. (Jan. 14, 2022), https://www.consumerreports.org/health/dna-test-kits/privacy-and-direct-to-consumer-genetic-testing-dna-test-kits-a1187212155/. A number of these recommendations were put forth by the Future of Privacy Forum. See *Privacy Best Practices for Consumer Genetic Testing Services*, Future Priv. Forum, (July 31, 2018) https://fpf.org/wp-content/uploads/2018/07/Privacy-Best-Practices-for-Consumer-Genetic-Testing-Services-FINAL.pdf.

